# Hydration-Mediated Energy Landscapes Govern Rotational Flexibility in Membrane-Bound Annexin V Assemblies

**DOI:** 10.1021/acs.nanolett.6c00388

**Published:** 2026-04-06

**Authors:** Ayhan Yurtsever, Kien Xuan Ngo, Takashi Sumikama, Ayaka Imamura, Shunsuke Mochizuki, Kaito Hirata, Haohui Zhang, Hiroki Konno, Kazuki Miyata, Takeshi Fukuma

**Affiliations:** † Nano Life Science Institute (WPI-NanoLSI), Kanazawa University, Kakuma-machi, Kanazawa 920-1192, Japan; ‡ Institute for Interdisciplinary Research in Science and Education (IFIRSE), ICISE, Quy Nhon 55131, Vietnam; § Laboratory of Biomolecular Dynamics at Nanoscale, Graduate School of Biostudies, Kyoto University, Yoshida-Konoe-machi, Sakyo-ku, Kyoto 606-8501, Japan; ∥ Center for Living Systems Information Science (CeLiSIS), Graduate School of Biostudies, Kyoto University, Yoshida-Konoe-machi, Sakyo-ku, Kyoto 606-8501, Japan; ⊥ Division of Frontier Engineering, 12858 Kanazawa University, Kanazawa 920-1192, Japan; # Department of Physical Science and Engineering, 12982 Nagoya Institute of Technology, Nagoya 466-8555, Japan; ∇ Division of Nano Life Science, 12858 Kanazawa University, Kanazawa 920-1192, Japan

**Keywords:** Annexin A5, Protein Hydration Shell, Interfacial Water Structure, Atomic Force Microscopy, Protein Assembly, Molecular Dynamics Simulations

## Abstract

Interfacial water organization and dynamics govern protein stability and function across molecular to supramolecular scales. Annexin V (AnxA5), a membrane repair protein, forms 2D assemblies on lipid membranes, yet the hydration role in repair remains unexplored. Combining three-dimensional atomic force microscopy (3D-AFM) and molecular dynamics (MD) simulations, we resolve the 3D hydration architecture of AnxA5 assemblies at molecular resolution. AnxA5 is enveloped by a continuous, nonlayered hydration network extending 1.5–2 nm into bulk solvent, exhibiting quasi-periodic lateral organization across crystalline and noncrystalline trimer domains. MD simulations indicate this network forms dynamic hydrogen-bonded bridges that may stabilize interdomain junctions, thereby modulating the local energy landscape. This hydration-dependent configurational flexibility, coupled with thermal fluctuations, drives stochastic, reversible trimer rotation, potentially modulating membrane interactions and Ca^2+^ coordination. Our findings establish interfacial water as a key mediator of supramolecular organization and stabilization, proposing a mechanism for hydration-mediated conformational flexibility during Annexin-driven membrane repair.

## Graphical abstract

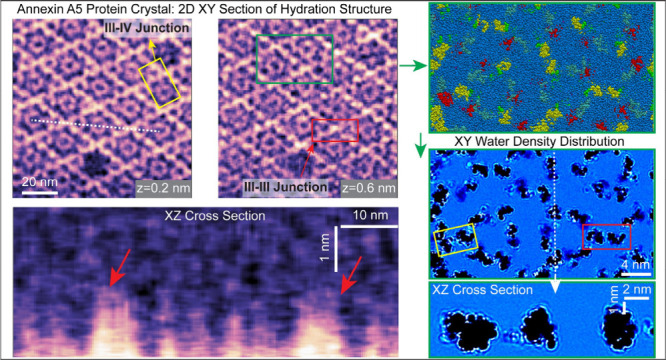

Hydration or solvation shells enveloping biological macromolecules possess structural and dynamical properties distinct from bulk solvent and play a critical role in governing molecular assembly, conformational stability, and function.^1–5^ These interfacial hydration shells stabilize folded protein architectures via hydrogen-bond networks with protein polar residues and dielectric electrostatic screening,^6^ and they mediate protein–protein and protein–ligand interactions by modulating binding affinity and selectivity,^7^ while imposing a dehydration barrier that proteins must overcome for effective biomolecular association.^8^ Beyond their structural role, these spatially structured solvent shells further govern critical biochemical processes, including protein folding,^9–11^ assembly,^12,13^ and crystallization,^3^ enzyme catalysis,^14^ molecular recognition,^15^ proton transfer, and transmembrane transport of water and ions.^16,17^ The synergistic dynamics between proteins and their hydration shells confer conformational flexibility, enabling adaptation to environmental perturbations through solvent-mediated structural reorganization that drives functional transitions and dynamics.^18–20^ The significance of water–protein interactions becomes even more pronounced in crowded cellular environments,^21–23^ as a large proportion of cellular water is organized within highly ordered hydration shells that encapsulate biomolecular surfaces and modulate desolvation energetics to govern the selectivity of intermolecular contacts,^6^ enabling specific binding while preventing nonspecific macromolecular aggregation.^24^ Despite their fundamental importance to biological function and assembly, the detailed molecular organization of protein-associated hydration shells remains a significant knowledge gap, mainly owing to the topological complexity and chemical heterogeneity of solvent-exposed functional groups. Characterizing these hydration patterns is crucial for decoding macromolecular interfacial recognition and interactions with protein arrays, enabling rational design of protein-based biosensors.^25^

Annexin V (AnxA5) serves as a robust model system to interrogate these solvation structures, as it forms highly ordered two-dimensional crystals upon binding to lipid membranes. Annexins are abundant cytoplasmic proteins that bind to negatively charged phospholipid membranes in a Ca^2+^-dependent manner^26^ and are abundantly expressed in diverse tissues in both intra- and extracellular compartments.^27^ They participate in various membrane-related biological processes, including exo- and endocytosis, regulation of molecular trafficking, membrane aggregation and dynamics, ion channel activity, and regulation of blood inflammation.^26,28,29^ Most notably, AnxA5 assembly represents a key component of cellular membrane repair machinery. During cellular injury, the influx of extracellular Ca^2+^ stimulates annexins to associate with membranes, which in turn triggers membrane repair processes.^30–32^ Although AnxA5–membrane association is known to be highly sensitive to local interfacial hydration environment,^33^ the molecular mechanisms governing this process—and their relevance to ordered protein array formation and membrane repair—remain poorly understood. Consequently, resolving the structure and dynamics of water at the AnxA5–membrane interface is essential to understand the molecular basis of AnxA5-mediated membrane repair.

Previously X-ray or NMR spectroscopy methods,^34,35^ neutron scattering,^36^ and terahertz spectroscopy,^37^ as well as sum frequency generation spectroscopy^38^ have been used to determine the structure and orientation of solvation shells around proteins. Although these techniques have contributed to probing certain aspects of the structure and dynamics of hydration shells around biomolecules, their reliance on ensemble averaging and restricted sensitivity to the first hydration shell have precluded a comprehensive understanding of biomolecular hydration. This bottleneck has left fundamental questions regarding the spatiotemporal extent of hydration gradients, interfacial layer thickness, and the nature of local density fluctuations—governed by specific protein domains or amino acid sequences—subjects of intense debate, underscoring the need for methodologies capable of resolving 3D local structural details of solvation layers at the molecular level.

Here, we used high-resolution three-dimensional atomic force microscopy (3D-AFM)^39^ to resolve the local 3D organization of water within the hydration shells surrounding AnxA5 2D protein crystals at CaCl_2_–AnxA5 interfaces. Complementary all-atom molecular dynamics (MD) simulations were carried out to substantiate and contextualize the experimental observations, providing molecular-level insights into the spatial organization and dynamic behavior of interfacial hydration shells.

It has been shown that AnxA5 forms 2D crystalline lattices with p3 and p6 symmetries on negatively charged phospholipid bilayers under aqueous conditions,^40–42^ with the lattice symmetry determined by the phosphatidylserine content of the membrane and the concentration of Ca^2+^ ions. X-ray crystallographic measurements have revealed that the tertiary molecular structure of AnxA5 consists of a central core composed of four structurally homologous α-helical domains, labeled I through IV, which are visually represented in different colors according to the description by Huber^43^ (Figure 1A,B), and a flexible N-terminal tail extending outward. The interdomain interactions give rise to two distinct modules; module 1 (domains I–IV) and module 2 (domains II–III). The domains within each module are noncovalently connected via the N-terminal tail, which bridges the two regions and extends toward the C-terminus. These interdomain interactions are mainly mediated by hydrophobic residues, whereas the contacts between paired modules (II–III) and (I–IV) are comparatively shorter and mainly stabilized by interactions between polar, charged protein residues.^43^ The planar 2D crystal lattice of AnxA5 consists of trimers arranged at hexagonal vertices in a 6-fold (p6) symmetric lattice (Figure 1C). The convex side of each trimer is oriented toward the lipid membrane, facilitating direct interactions that mediate membrane binding and contribute to stabilization of the lattice architecture. Concurrently, the flexible N-terminal region is exposed at the surface, where it dynamically interacts with the surrounding environment and plays a crucial role in defining molecular recognition characteristics and modulating macromolecular interactions and assembly. In the 2D p6-symmetric lattice, each AnxA5 trimer contacts neighboring trimers via domain III residues (yellow/orange), forming intermolecular interactions that stabilize the assembly (Figure 1C).

**Figure 1:**
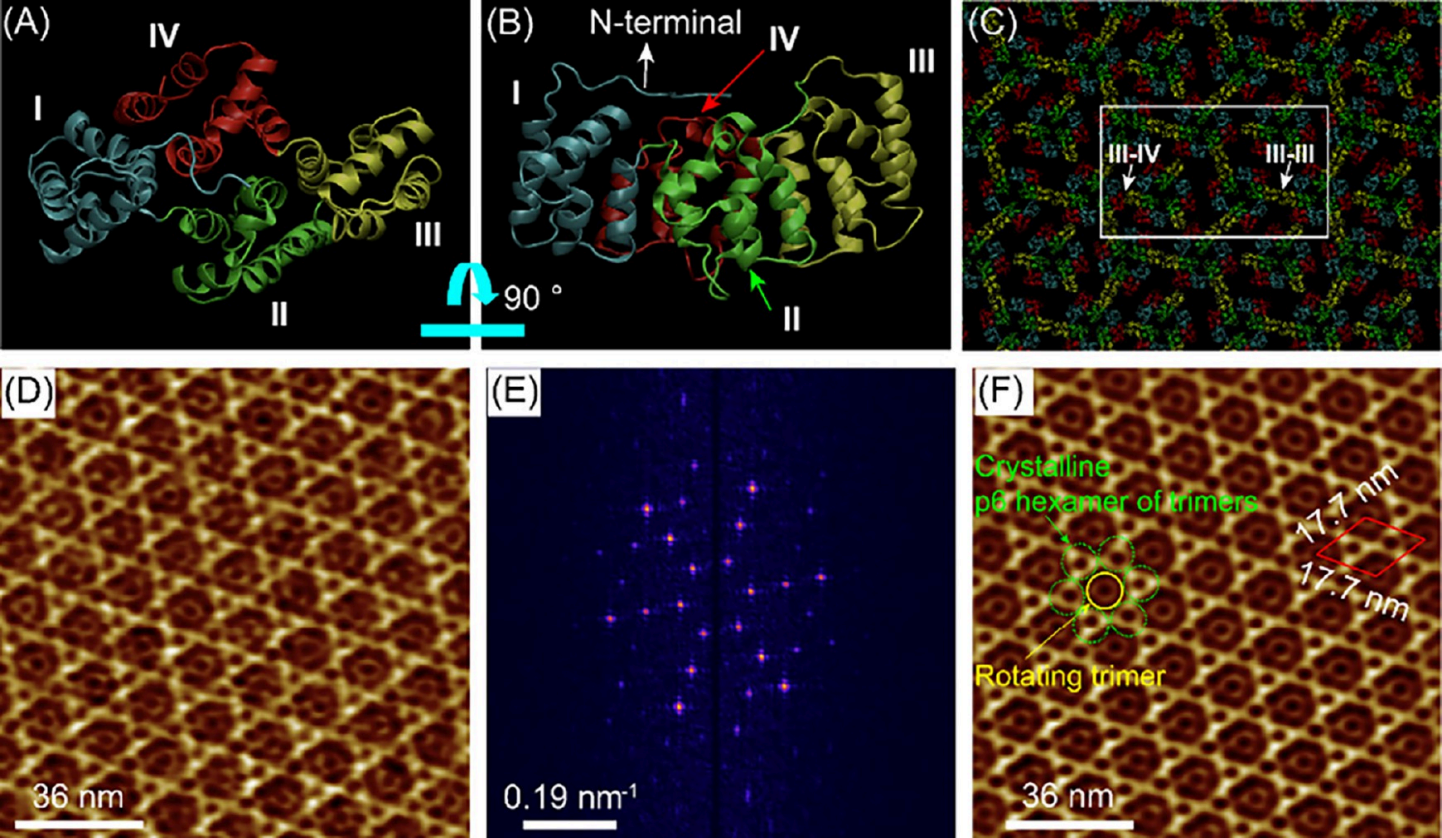
3D molecular structure of AnxA5 (ribbon diagram), top (A) and side (B) views, respectively. AnxA5 comprises four structurally homologous α-helical domains (I–IV) forming a central core and a flexible N-terminal region. The membrane-binding surface is convex, while the N-terminal regulatory domain lies on the concave face, separated from the Ca^2+^-binding loops on the opposite side. (C) Structural arrangement of AnxA5 molecules within the crystal lattice formed on a lipid membrane. (D) High-resolution AFM image of the 2D AnxA5 crystalline array, obtained in the presence of 2 mM Ca^2+^. (E) 2D FFT spectra obtained on the image shown in panel D. (F) FFT reconstructed AFM image obtained by executing inverse FFT. The lattice consists of hexameric assemblies of trimers organized in a honeycomb arrangement (green circles), with the central non-p6 trimer (yellow circle) displaying rotational freedom. Lattice constants of the p6 crystal structure are ***a*** = ***b*** = 17.7 nm; ***γ*** = 120°. The unit cells are indicated with a red lozenge pattern.

Figure 1D–F shows typical high-resolution AFM images of the AnxA5 2D crystals formed on a lipid membrane supported on mica. Large-area AFM images (Figure S1) show that AnxA5 assembles into crystalline lattices composed of domains with distinct orientations, separated by grain boundaries of reduced molecular order. A well-ordered 2D crystalline lattice of AnxA5 was resolved in AFM images, consistent with previous reports of its hexagonal symmetry and trimeric organization. In agreement with the previous AFM studies,^44^ we found that AnxA5 trimers (Figure 1D-F) assembling in a lattice with p6 symmetry. The central noncrystalline trimers—appearing as circular features—engage with neighboring p6-crystalline units at specific angular orientations. Such interactions reflect the rotational degrees of freedom and the dynamic reorientation of these trimers within the lattice architecture (Figure 1D,F).

To bridge the structural organization of AnxA5 assemblies with their functional role in membrane repair, we investigated the 3D molecular arrangement of interfacial water near the AnxA5 2D crystal surface using state-of-the-art 3D-AFM, thereby revealing insights into hydration-mediated protein assembly and stability essential to its repair function. This technique has previously been employed to resolve solvation structures at atomic and molecular scales across various substrates and interfaces,^45,46^ including solid^47^ and soft samples,^48,49^ as well as structural biopolymers.^50,51^ A representative 3D map of the AnxA5-solvent interface is presented in Figure 2A, showing the spatial variations of interfacial hydration structures near the crystal surface within the 3D interfacial space. The 2D XY in-plane slices within the hydration zone, extending approximately 1.5–2 nm from the surface, reveal highly ordered hydration structures that recapitulate the symmetry and molecular organization of the underlying AnxA5 2D crystal lattice (Figure 2B-i–vi; Figure S2). These structured hydration features gradually fade away with increasing distance, seamlessly transitioning into bulk solvent. The vertical 2D maps across different directions exhibit quasi-periodic density modulation with a lateral spacing of approximately 5–6 nm (Figure 2C–E), closely corresponding to the dimensions of an AnxA5 monomer composed of four domains (I–IV), measuring approximately 64 × 40 × 30 Å, and reflecting the spatial arrangement of AnxA5 trimers within a single 17–18 nm repeat unit.

**Figure 2:**
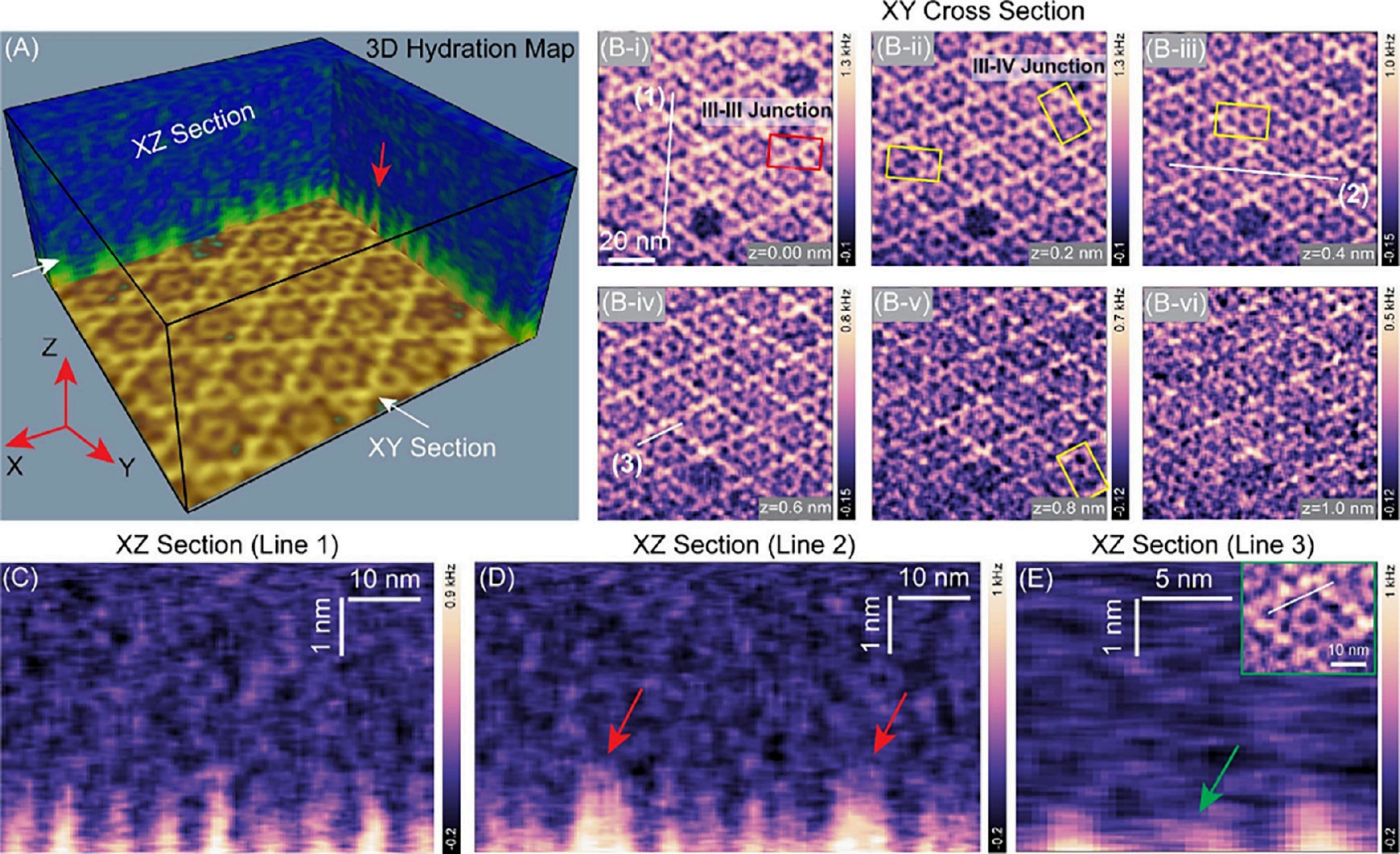
3D organization of interfacial water surrounding the AnxA5 2D crystal interface. (A) The 3D map of the interfacial water structure, demonstrating the extraction of 2D XZ and YZ cross-sectional slices. (B-i to B-vi) 2D planar views of water organization at sequential heights above the 2D AnxA5 crystal. (C–E) Vertical 2D slices along lines (1), (2), and (3) show highly localized water molecules arranged in a quasi-periodic lateral pattern with periodicity of ∼5–6 nm, extending approximately 1.5 nm from the crystal surface. Panel (E) emphasizes the formation of hydration structures bridging the central trimers and adjacent trimers within the crystal lattice. Inset: XY cross-section, with the white line denoting the location of the corresponding 2D profile. Green arrows and yellow rectangles highlight hydration structures linking crystalline and noncrystalline AnxA5 trimers at the domain III–IV junction, whereas red arrows and rectangles denote hydration bridges between crystalline trimers at the domain III–III junction.

The observed molecular organization of water is very different from that of highly ordered hydration structures on solid surfaces, such as, mica, HOPG, and other crystalline interfaces.^50,52^ On solid substrates, water molecules organize into discrete hydration layers exhibiting oscillatory density profiles along the surface normal. On hydrophobic substrates, these layers exhibit reduced local density near the surface and remain unanchored to specific lattice atoms,^53,54^ allowing them to maintain a shell-like structure. In contrast, more intricate hydration patterns emerge near amphiphilic structural biopolymers, where localized, exclusive interactions with specific functional sites dictate the interfacial water organization.^51^ Rather than exhibiting domain-specific water organization, the AnxA5 monomer is uniformly enveloped by a continuous hydration layer covering all domains. This likely arises from the conformational flexibility of the N-terminal domain (Supplementary Videos 1 and 2), whose fluctuations extend across the protein surface; the surrounding water molecules dynamically track these motions, resulting in a cohesive solvent interface that spans the entire molecular unit. The force–distance curves taken through hydration structures exhibit a predominantly repulsive force profile. The effective range of this hydration-mediated repulsion extends approximately 1.5–2 nm (Figure S3). To clarify the physical origin of this signal, we evaluated the decay characteristics of the 3D-AFM force profiles (Figure S4). Exponential fitting of the nonoscillatory repulsive branch, *F­(d)* = *F*_0_e^*–d/λ*^, yields a decay length of ∼0.3 nm (0.37 ± 0.28 nm), comparable to the thickness of a single hydration layer. The force attenuates within ∼1.0–1.2 nm, consistent with the hydration region and much shorter than the ranges typically associated with electrostatic or van der Waals interactions, indicating that the measured force pattern mainly reflects short-range hydration forces.^55^ At the interface between neighboring AnxA5 trimers within the p6 crystalline lattice (red rectangle in Figure 2B-i), the hydration network displayed a pronounced increase in both localization (brighter contrast) and spatial extension, suggesting increased water organization at this intermolecular junction (red arrows, Figure 2D). This enhanced hydration likely reflects a solvent-accessible, dynamically flexible region that stabilizes trimer–trimer interactions essential for AnxA5 lattice assembly and maintenance, while orchestrating dynamic reorganization to enable adaptive responses during membrane remodeling and repair.

Notably, we also observed hydration-mediated molecular interactions between two AnxA5 trimers—the central noncrystalline AnxA5 trimer and the crystalline AnxA5 trimer—positioned at specific angular orientations (see yellow rectangles in Figure 2B-i–vi; Figure S5). As shown in Figure 2E, the 2D vertical map along the white line (see inset) reveals a hydration structure bridging two trimers through hydration-induced hydrogen bonds. This structure appears relatively weaker and shorter in extent than the hydration observed over the crystalline p6 domains. Previous studies have shown that central, or non-p6, trimers exhibit dynamic rotational motion arising from weak lateral interactions with neighboring crystalline protein trimers.^56^ This rotational flexibility is thought to enable structural rearrangement of the lattice in response to membrane stress or local ionic fluctuations, thereby contributing to annexin-mediated membrane remodeling and functional assembly. The role of this interfacial water in facilitating such rotational transitions is elaborated below through analysis of the hydration-mediated interfacial energy landscape.

We further characterized the spatial organization of interfacial hydration at lattice vacancies—i.e., structural defects resulting from the absence of central noncrystalline trimers at 6-fold symmetry centers (Figure 3). These vacancies, which appear as dark depressions in AFM images, allowed us to probe how local structural inhomogeneities determine the spatial distribution of the hydration layer (Figure 3A-i–iv). Our observations revealed that a highly ordered hydration network emerges above the regular AnxA5 lattice domains; however, this structuring is notably absent or weakly fluctuating in a disordered form at the hollow vacancy sites (Figure 3B-i–iii). The lack of a structured hydration layer at these vacancy regions suggests a distinct local solvation environment. Compared to the quasi-periodic hydration network observed over the crystalline domains, the weakly ordered water at vacancies implies a reduced desolvation penalty, which may facilitate the adsorption of external proteins or biomolecules. This interpretation is consistent with previous investigations of streptavidin (SA) protein assemblies on AnxA5 crystals,^57^ where SA molecules exhibited preferential accumulation at hollow vacancy sites—an effect plausibly linked to the lower energetic cost of displacing water in regions lacking structural coherence. Consequently, the observed modulation of hydration structuring near lattice discontinuities suggests that local variations in the hydration landscape may regulate biomolecular adsorption and nucleation phenomena.

**Figure 3:**
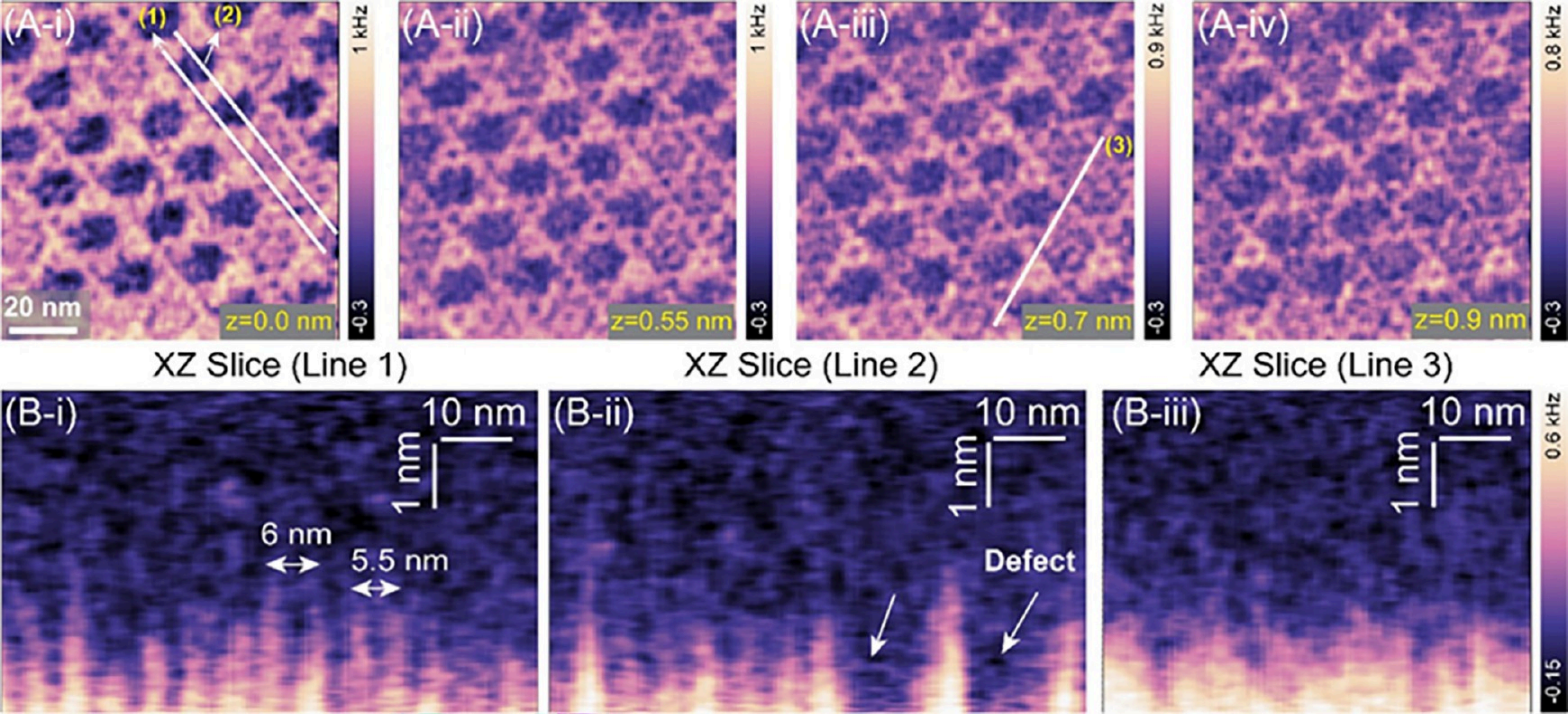
Interfacial water organization above AnxA5 lattice vacancies at the central trimer. (A-i—iv) Representative XY slices extracted from the 3D hydration map, illustrating the lateral organization of interfacial water at varying distances (z = 0 to 0.9 nm) from the AnxA5 lattice with central trimer vacancies. The dark depressions correspond to missing central AnxA5 trimers in the lattice. (B-i–B-iii) Corresponding 2D vertical cross-sectional profiles acquired along the paths marked with numbers (1), (2), and (3), revealing hydration architectures over crystalline domains and lattice vacancies. The interfacial region adjacent to the crystalline AnxA5 domains displays a quasi-periodic arrangement of localized hydration domains with a characteristic lateral periodicity of ∼ 5–6 nm, whereas water above the vacancy sites lacks discernible structural ordering. In panel B-iii, the interfacial water along line 3 exhibits continuous hydration features lacking lateral molecular organization.

In contrast, a continuous hydration architecture is observed along line 3 (white line in Figure 3A-iii), which traverses the hexagonal zigzag boundary across crystalline trimers with close interdomain contacts (Figure 3A-iii and B-iii). Along this path, we observed a vertically extended solvent column with no detectable lateral molecular ordering. This feature emerges from the solvent-exposed concave face, where relatively small interdomain distances enable N-terminal tail fluctuations to overlie the protein domains. This promotes strong water interactions while preventing ordered localization and maintaining a vertically continuous, dynamically fluctuating hydration column.

In order to rationalize the experimental hydration profiles obtained through 3D-AFM measurements, MD simulations were conducted to provide an atomic-level understanding of the interfacial water–protein interactions and the resulting density distributions. We constructed a supramolecular assembly comprising 18 AnxA5 monomers (PDB: 6K22) in a p6 symmetric lattice,^29^ which was then solvated by 241,548 water molecules, 780 Na^+^, 70 Ca^2+^ (including 54 Ca^2+^ ions preserved from the crystal structure), and 656 Cl^–^, detailed in the Methods section (Figure 4A, Figure S6, Supplementary Videos 1 and 2). The 3D water density derived from MD simulations reveals pronounced water structuring both laterally between adjacent protein trimer domains and vertically from the surface (Figure 4B–D, Figures S7–S9, Supplementary Videos 3–6), highlighting the key role of interfacial water in mediating interactions, stabilizing assemblies, and enabling molecular recognition.^58^ The simulated water density distribution, analyzed in both planar and vertical dimensions, closely replicates the experimentally observed hydration features. In particular, the quasi-periodic arrangement of water density observed in the AFM measurements with repeating of 5–6 nm is in good agreement with the MD simulations of water density (Figure 4B). The distribution of water oxygen density mainly surrounds AnxA5 trimer domains in vertical and lateral directions; 2D vertical maps reveal discrete molecular organization along certain lateral paths (Figure 4B, Line 1), in contrast to a relatively continuous lateral layering (Lines 2 and 3, Figure 4Di–ii). While experimental and simulated hydration structures are qualitatively consistent, the hydration force range measured by 3D-AFM (1.5–2.0 nm) exceeds the ∼1.0 nm density oscillation range predicted by MD simulations. This discrepancy arises mainly from the absence of the probe and its hydration shell in MD models; 3D-AFM captures the convolution of tip and surface hydration layers, extending the apparent range.^59^ Additionally, thermal fluctuations and probe dynamics broaden the AFM signal compared to the sharper density transitions observed in MD simulations.

**Figure 4:**
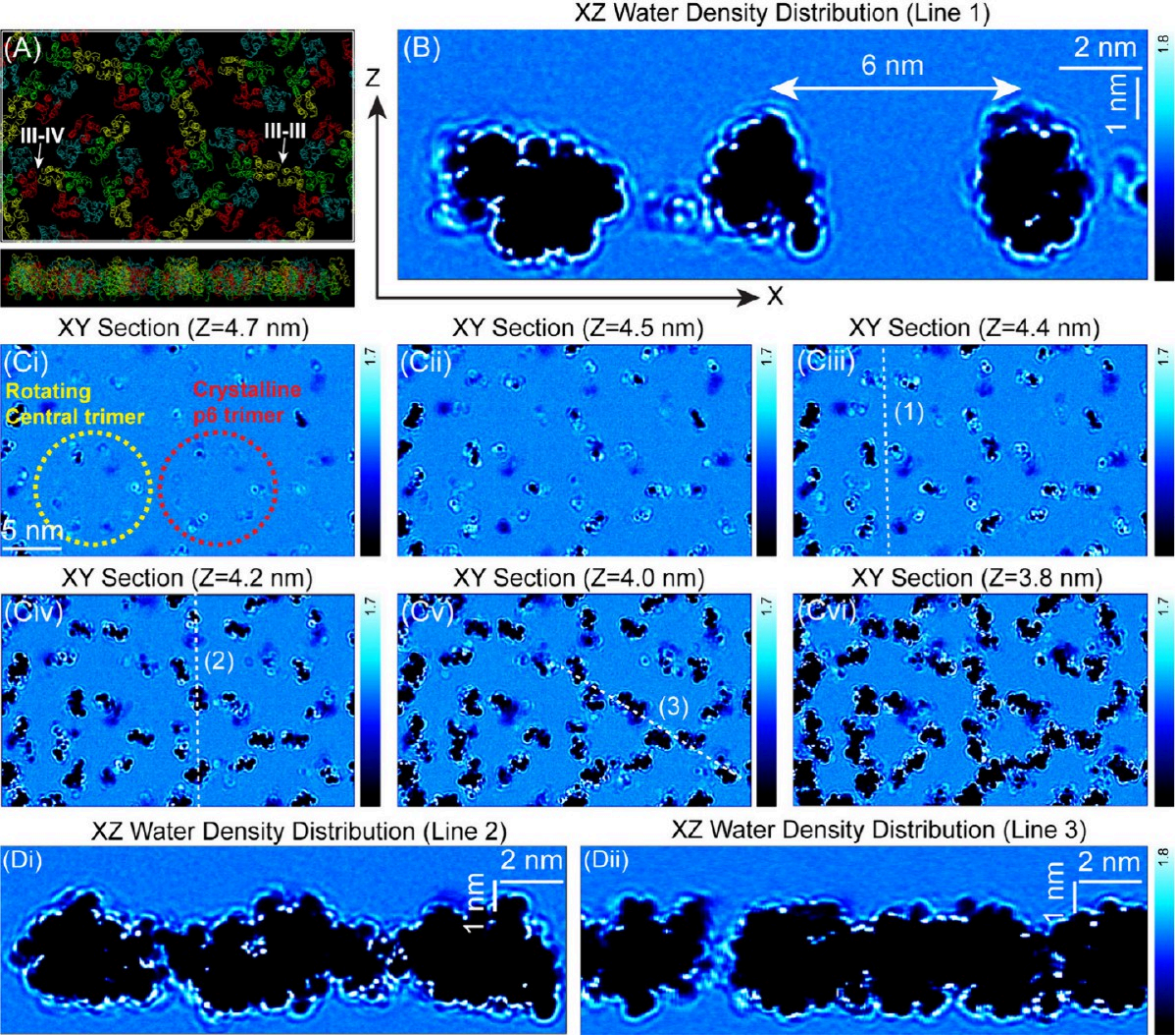
Spatial distribution and local organization of water oxygen density at the AnxA5–membrane interface. (A) Simulated supramolecular architecture of AnxA5 2D arrays on the lipid membrane interface (top view) with its corresponding cross-sectional molecular arrangement (bottom). (B) 2D vertical water oxygen density map extracted along the white dashed line (1) in panel (C-iii), showing discrete density variations. (C-i–vi) 2D lateral (XY-plane) maps of water density corresponding to successive hydration layers at z = 4.7, 4.5, 4.4, 4.2, 4.0, and 3.8 nm, respectively. (D-i,ii) 2D vertical water density distributions acquired along lines (2) and (3), respectively. The darker regions correspond to the AnxA5 assembly structure.

The hydration-mediated molecular interactions observed between adjacent trimers—specifically, a central noncrystalline trimer and its neighboring crystalline counterpart—at defined angular orientations (Figure 2E, inset), as well as between crystalline–crystalline trimers, are further substantiated by MD simulations, revealing that lateral water structuring at interfacial junctions mediates the coupling between adjacent trimers (Figure 5A,B). At certain interfacial separations, the hydration shells of adjacent trimers overlap laterally (Figure 5C-i–iii), forming an interconnected hydrogen-bonded network of bridging water molecules. This overlap establishes a spatially coherent hydration bridge across neighboring protein domains (Figure 5D,G), as evidenced by discrete multiple density peaks (red arrows) in the 1D vertical density profiles taken from both crystalline and crystalline–noncrystalline interfaces (Figures 5E,F and 5H,I). This suggests that bridging hydration layers effectively extend the reach of protein interfaces and mediate hydrogen bonding and molecular recognition even at relatively large separations—where direct residue contacts are absent or sterically/geometrically inaccessible. Such hydration-mediated interactions can lower or modify the certain local energetic barriers between different configurations, enabling the central domains, aided by thermal energy, to rotate between energetically metastable states in a stochastic, stepwise manner (Figure S10, Supplementary Video 7). The proximal residue distances at the junctions between neighboring trimers (Figures S11–S13) support a contribution from water-mediated interactions. The residues Thr215, Ile216, Ser217, and Phe180 in domain III of AnxA5 are positioned at the interdomain interface and likely contribute to stabilizing interactions between adjacent crystalline trimer.^28,57^ Within the 2D lattice, inter-residue separations span ∼ 3–12 Å (predominantly 6–12 Å), with closest Ile216–Ile216 contacts (∼3 Å) and Phe180–Phe180 interactions (∼5 Å) consistent with π–π stacking (Figures S11 and S12, Supplementary Videos 8 and 9). Other residue pairs fall within ∼ 9–12 Å; complete inter-residue distance data are provided in Figures S11–S13.

**Figure 5:**
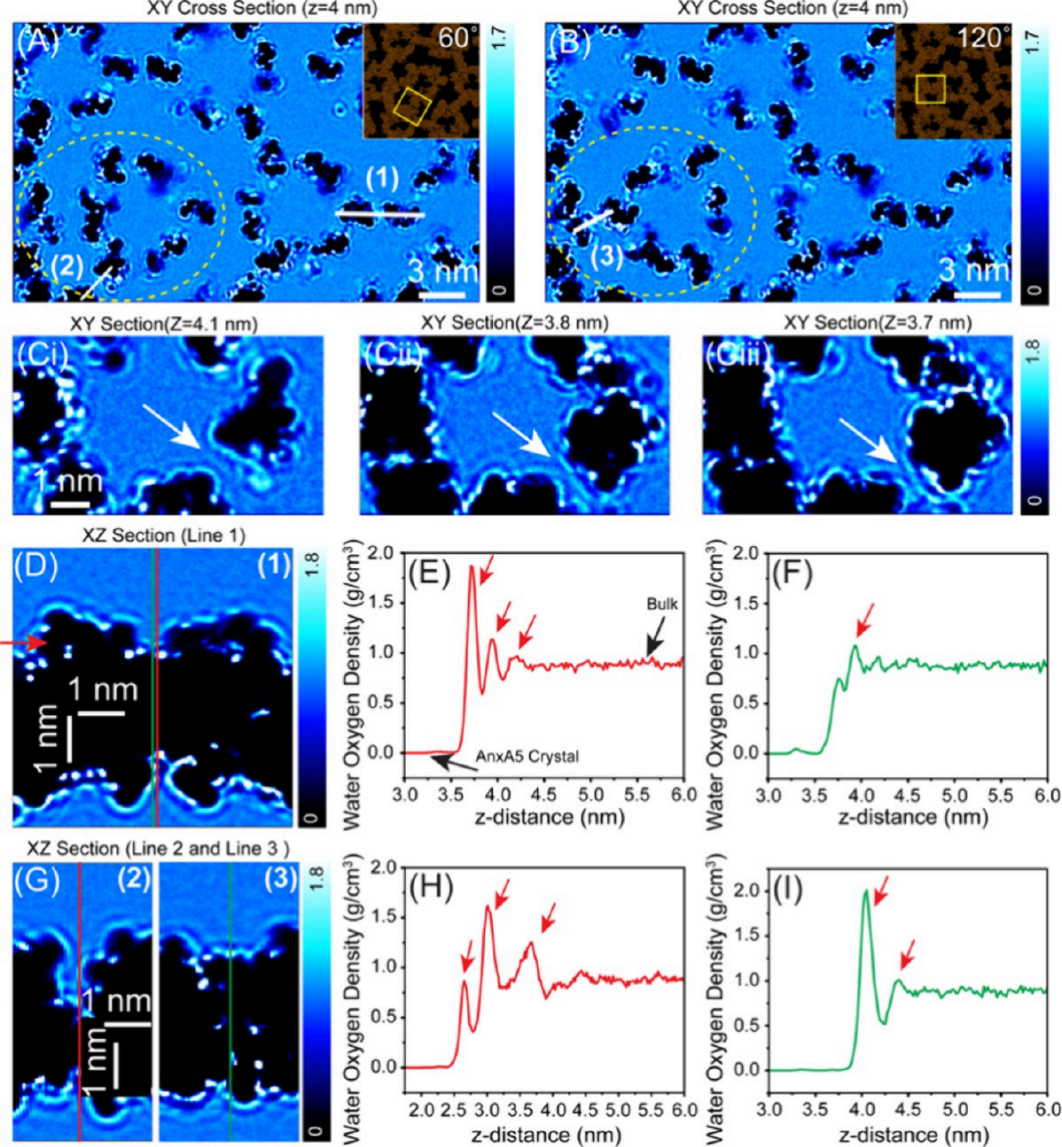
Water density distributions at crystalline (III–III) and crystalline–noncrystalline (III–IV) junctions at 60° and 120° rotations. (A, B) XY sections extracted from the 3D density map at z = 4 nm along the red arrow shown in (D), corresponding to 60° and 120° rotations of the central trimer, respectively. (C-i–iii) Zoom-in views of the XY slices reveal the detailed boundary structure at the III–IV interface, with white arrows highlighting overlapping regions of ordered high-water density. (D) XZ slice along junction (1) between domain III–III crystalline trimers, showing vertical water organization. (E, F) 1D density profiles along the red and green lines in (D), respectively. (G) XZ slices along junctions (2) and (3) between domain III–IV of noncrystalline and crystalline trimers, highlighting water organization at the interfaces. (H, I) Corresponding 1D density profiles. Continuity of hydration shells across the junctions is evident in the 2D maps and corroborated by localized peaks of high-water density in the 1D density profiles (red arrows).

On the other hand, the interfacial interactions between neighboring crystalline and noncrystalline p6 trimers are mainly mediated by domain III and IV, as these domains are positioned in close contact within the assembled structure (red and orange in Figure 1C). In particular, residues Thr215, Ile216, Ser217, and Gly218 in domain III, and Ser295, Tyr297, and Ser298 in domain IV have been reported to contribute to intertrimer contacts and stabilization.^28,57^ Analysis of six representative junctions between the rotating noncrystalline trimer and adjacent crystalline trimers reveals minimum inter-residue separations ranging from 3 to 12 Å, with Gly218–Ser298, Gly218–Tyr297, and Ser217–Tyr297 pairs exhibiting consistently short distances of 5 Å or less (Figure S13). Given that distances exceeding ∼6 Å are beyond the range of direct hydrogen bonding, the observed lattice stability may involve bridging water molecules. These solvent-mediated linkages can facilitate hydrogen-bonding networks across domain III–III and domain IV–III interfaces.

We quantified the translational mobility of interfacial water via diffusion coefficients (*D*) calculated at the III–III and III–IV junctions using the SPC/E water model.^60^ Our analysis reveals substantially attenuated water dynamics at these interfaces relative to the bulk phase. Specifically, the III–III interface exhibited a *D* of 1.09 × 10^–5^ cm^2^/s, while the III–IV interface displayed slightly higher mobility at 1.26 × 10^–5^ cm^2^/s (computed for ensembles of 300 water molecules). These values represent a marked retardation of solvent mobility relative to the simulated and experimental bulk diffusion coefficients of 2.49 × 10^–5^ cm^2^/s and 2.30 × 10^–5^ cm^2^/s, respectively, highlighting trimer–trimer junction-specific confinement effects on local solvent kinetics. The higher water mobility at the III–IV junction implies a more flexible, loosely coupled interface. In the context of biological membranes, such conformational flexibility facilitates adaptation of the protein lattice to membrane curvature and supports structural rearrangements required for functional assembly.

High-speed atomic force microscopy studies have previously demonstrated that the noncrystalline central trimer can exhibit rotational freedom under specific conditions.^56^ Compared to other trimers, it is less tightly incorporated into the AnxA5 2D lattice and alternates between two preferred orientations separated by 60° (Supplementary Video 7). These rotational dynamics arise from weak intermolecular interactions, which allows the central trimer to stochastically and rapidly transition between equivalent orientational configurations without encountering a significant energy barrier.^56^ Despite these observations, the molecular origins of these rotameric motions and their coupling to the interfacial hydration shell remain poorly understood. Based on the observed interfacial hydration structures, we propose that hydration at the domain III–IV junctions may contribute to shaping the energetic landscape governing trimer rotation. In particular, structured water molecules observed at these interfaces may mediate bridging interactions between neighboring residues across the interdomain junctions, potentially modulating the local stabilities and facilitating stochastic rotational transitions.

To quantify the impact of hydration, we evaluated the energetic differences across three distinct domain III–IV junction configurations in both the presence and absence of explicit water (Figures 6A–C). Initially, stable primary contacts at junction 1, relative to weaker trimer–trimer interactions at junctions 2 and 3—differing in orientation by ∼ 60° from the primary contact—introduce anisotropy in the energy landscape. This anisotropy, coupled with thermal fluctuations and dynamic reorganization of the hydrogen-bonding network, facilitates rotational transitions by reducing energetic differences between distinct junction configurations. Consequently, transient weakening of primary contacts permits trimer rearrangement, promoting its rotation toward neighboring crystalline trimers. As shown in Figures 6B,C and Figures S14–S16, the energy differences between junction 1 (green) and junction 2 (dark yellow) configurations—initially distinct due to differences in the number of close contacts between residues (Figure 6D)—significantly decrease and eventually converge upon water inclusion. This indicates that hydration may modulate the local interaction energetics at the domain III–IV junctions, potentially facilitating transitions between distinct junction conformations. While the present analysis does not explicitly determine the energetic barriers associated with junction formation, the MD-derived interaction energies provide a basis for comparing the relative stability of different junction configurations.

**Figure 6:**
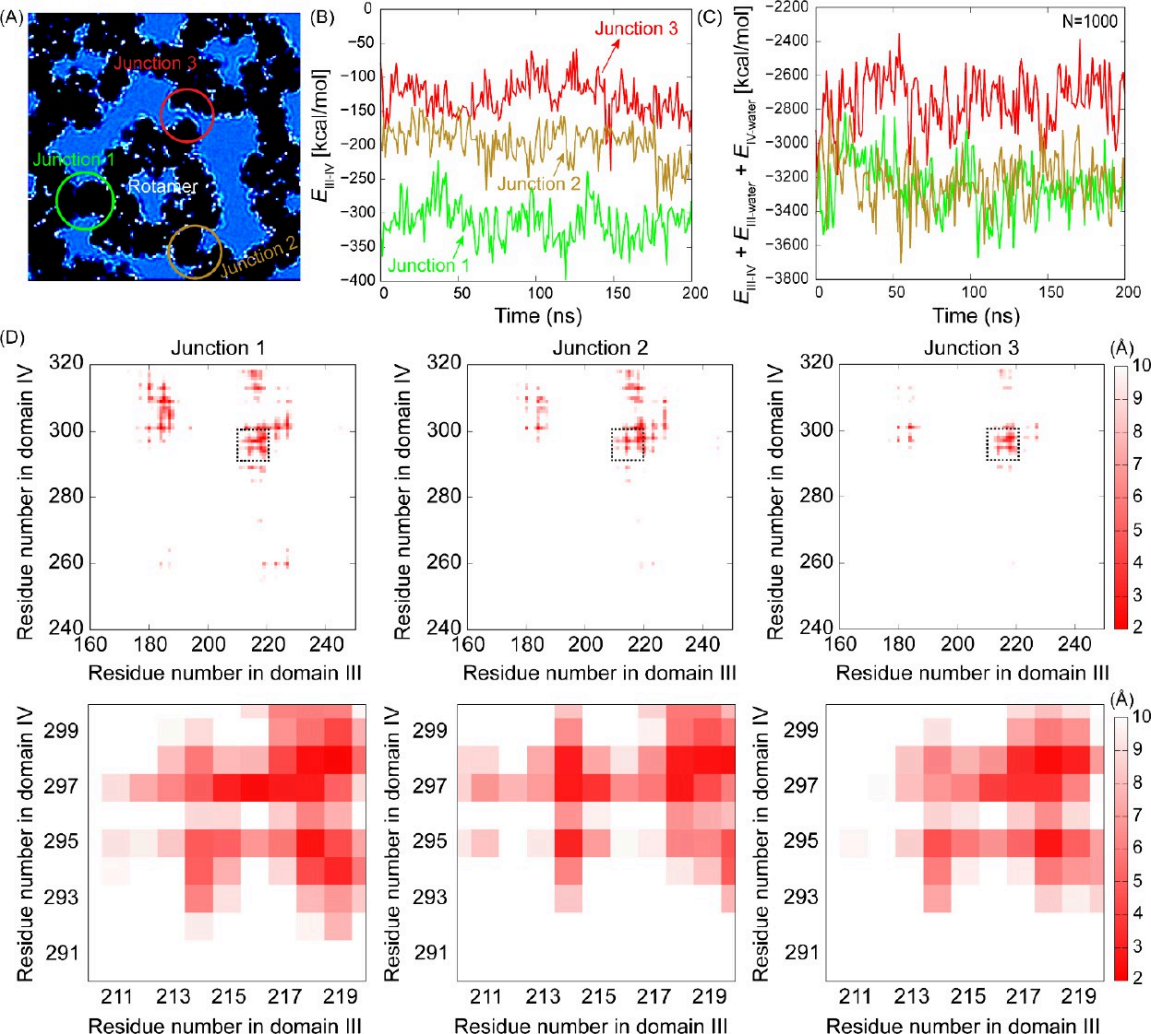
Interaction energies and interdomain distance distributions at the III–IV junctions. (A) 2D crystalline structure of AnxA5 illustrating the three junctions formed between domains III and IV. (B) Interaction energies between domains III and IV of the three junctions determined without water, *E*_III–IV_. (C) Interaction energies of the same junctions in the presence of water molecules at the interface. *E*_III‑water_ and *E*_IV‑water_ denote the interaction energies between domain III and 1000 interfacial water molecules and between domain IV and the same water molecules, respectively. The energy differences between the green and dark yellow junction configurations decrease and eventually vanish upon the inclusion of water, indicating that hydration effectively minimizes the energetic barrier separating distinct junction conformations. In panels (B, C), “*E*” represents the total nonbonding interaction energy, calculated as the sum of electrostatic and van der Waals interactions derived from the MD simulations. (D) Distribution of minimum distances between domains III and IV across junctions 1–3. The upper panels show all residue pairs. The lower panels highlight selected pairs within the dashed black box in the upper panels, corresponding to those shown in Figure S13.

In summary, this study provides a high-resolution 3D map of the hydration structure surrounding the AnxA5 supramolecular assembly. We demonstrate that AnxA5 is enveloped by a 1.5–2 nm thick, quasi-periodic hydration shells that bridge protein–protein domain interfaces. A fundamental characteristic of this interface is its continuous, nonlayered organization, linked to the chemical heterogeneity and conformational flexibility of protein surfaces. Notably, the absence of discrete, domain-specific hydration shells reveals profound spatiotemporal coupling between the flexible N-terminal ensemble and surrounding water molecules. Rather than a passive solvent layer, this dynamic interfacial architecture functions as an active energetic modulator that reshapes the energy landscape, facilitating rotational degeneracy and switching of trimeric units essential for lattice adaptability, thereby supporting a molecular model for the conformational transitions underlying Annexin-mediated membrane repair. These observations underscore the potential significance of solvent structuring in modulating protein assembly dynamics; however, establishing whether continuous hydration networks constitute a general and intrinsic feature of protein interfaces will require systematic 3D-AFM investigations across a broader spectrum of structurally diverse proteins.^61^

## Supplementary Material























## Notes

Materials and methods, supporting figures, STA-derived force and decay-length maps, structural details of the simulated AnxA5 model, time-dependent analyses of proximal distances for residues involved in domain III–III and III–IV interactions, and interaction energy profiles as a function of interfacial water content, including first-shell hydration only (PDF)
Side view of AnxA5, highlighting the dynamically fluctuating N-terminal domains (MP4)
Side view of AnxA5, Side view of AnxA5, highlighting the C-terminal domains (MP4)
Interfacial water density distribution near the AnxA5 protein assembly at a 30 ns simulation window (MP4)
Interfacial water density distribution near the AnxA5 protein assembly at a 200 ns simulation window (MP4)
Cross-sectional and top views of the assembled AnxA5 crystal with interfacial water organization (MP4)
Water oxygen density distribution across the AnxA5 assembly with distinct color coding (MOV)
Rotational motion of the central non-crystalline p6 trimer in the 2D AnxA5 assembly (MP4)
Visualization of the interfacial contact region between neighboring domain III–III interfaces (MP4)
Visualization of the interfacial contact region between neighboring domain III–III interfaces (MP4)
Descriptions of Videos 1–9 (PDF)
