# Supplementary material for: Hydration-Mediated Energy Landscapes Govern Rotational Flexibility in Membrane-Bound Annexin V Assemblies
Source: Nano Lett. 2026 Apr 6;26(14):4719–29. doi: 10.1021/acs.nanolett.6c00388 (PMC13088367; doi:10.1021/acs.nanolett.6c00388)
Supplement: Supplementary file 1 [file nl6c00388_si_001.pdf]

Supporting Information for

Hydration-Mediated Energy Landscapes Govern Rotational Flexibility in Membrane-Bound Annexin V Assemblies

Ayhan Yurtsever[†,*], *Kien Xuan Ngo*[†,‡], *Takashi Sumikama*[†,‖,#,*], *Ayaka Imamura*[†], *Shunsuke Mochizuki*[¶], *Kaito Hirata*[§], *Haohui Zhang*[††], *Hiroki Konno*[†], *Kazuki Miyata*[†,¶,††,*], *Takeshi Fukuma*[†,¶,††,*]

[†]Nano Life Science Institute (WPI-NanoLSI), Kanazawa University, Kakuma-machi, Kanazawa, 920-1192, Japan

[‡]Institute for Interdisciplinary Research in Science and Education (IFIRSE), ICISE, Quy Nhon, 55131, Vietnam

[‖]Laboratory of Biomolecular Dynamics at Nanoscale, Graduate School of Biostudies, Kyoto University, Yoshida-Konoe-machi, Sakyo-ku, Kyoto 606-8501, Japan

[#]Center for Living Systems Information Science (CeLiSIS), Graduate School of Biostudies, Kyoto University, Yoshida-Konoe-machi, Sakyo-ku, Kyoto 606-8501, Japan

[¶]Division of Frontier Engineering, Kanazawa University, Kanazawa 920-1192, Japan

[§]Department of Physical Science and Engineering, Nagoya Institute of Technology, Nagoya 466-8555, Japan

[††]Division of Nano Life Science, Kanazawa University, Kanazawa 920-1192, Japan

*E-mail: yurtsever@staff.kanazawa-u.ac.jp; fukuma@staff.kanazawa-u.ac.jp;

k-miyata@staff.kanazawa-u.ac.jp; takashi.sumikama@gmail.com

This PDF file Includes:

1. Materials and Methods
2. Supplementary Figures

   Figures S1-S16
3. Supplementary Movies

   Supplementary Videos 1-9
4. References

1. Materials and Methods

2D AnxA5 crystal preparation

Annexin A5 (AnxA5) 2D-crystals were formed on supported lipid bilayers by addition of AnxA5 to preformed membrane patches on Mica. The AnxA5 proteins expressed in *E. coli* cells were purified as described in ref.[1]. A thawed aliquot of AnxA5 was ultracentrifuged (Himac CS100GXL, Hitachi, 80,000 rpm, 5 minutes, 4°C) to remove aggregated AnxA5. The clarified supernatant was carefully recovered and AnxA5 protein concentration was measured by a protein assay kit (Bio-Rad Protein Assay Dye Reagent Concentration, BIO-RAD). 2D AnxA5 crystal was grown on the lipid bilayer containing DOPC:DOPS:biotinyl-DOPE 7:2:1 (wt/wt/wt) in crystal buffer (buffer C) containing 150 mM NaCl, 2 mM $CaCl_2$, and 10 mM HEPES-NaOH (pH 7.4) at room temperature. Buffer C was sterile filtered using a sterile syringe filter (CA 0.22 μm, AS ONE Corporation, Japan). Briefly, 1 mg/ml liposomes composed of the above lipid mixture were made in buffer C as reported previously[2]. The liposomes were diluted in buffer C to a final concentration of 0.1 mg/ml. 100 μl diluted liposomes were gently applied on a cleaved mica surface (mica with a diameter 12 mm) and incubated at room temperature for ~2 h or longer for forming the lipid bilayer. The lipid surface was further cleaned to remove lipid aggregates or intact liposomes by gently exchanging with buffer C (100 μl buffer C for each time, repeated 3-4 times). Next, AnxA5 protein was diluted in buffer C to a final concentration of 1.5 μM and gently deposited 100 μl this diluted AnxA5 solution onto lipid bilayer, and incubated for ~15-30 min at room temperature to form 2D AnxA5 crystal. The formed crystal surface was further cleaned by gently exchanging with buffer C (100 μl buffer C for each time, repeated 3-4 times), prior to FM-AFM investigation.

FM-AFM Setup

The interfacial structural organization at the AnxA5 crystal–water interface was investigated using a custom-built frequency modulation atomic force microscopy (FM-AFM) system

operated in liquid environments and equipped with an ultra-low-noise cantilever deflection sensor.[3] The oscillation of the AFM cantilever was driven by photothermal excitation with an infrared laser beam with a wavelength of 785 nm. Scanning control was implemented via a commercial AFM controller (ARC2, Asylum Research), while the oscillation amplitude was regulated by an external control unit (OC4, SPECS, Germany) to maintain a constant amplitude during imaging. The AFM was operated in constant frequency shift (Δf) mode, where the tip-sample distance is adjusted such that Δf is kept constant. AFM measurements were performed using 160AC-NG cantilevers (OPUS) with a nominal spring constant of 26 N/m and a tip radius smaller than 8 nm and NCHAuD cantilevers with a nominal spring constant of 42 N/m and a tip radius smaller than 10 nm. The spring constant of each cantilever was experimentally determined using the standard thermal tuning method. AFM image rendering and data processing were performed by using the WSxM and Gwyddion image analysis software.

3D-AFM Measurements

The 3D force or Δf maps at the AnxA5 crystal-buffer interface were generated using the 3D-AFM force mapping approach developed by Fukuma *et al.*[4] This method involves systematically scanning the AFM tip both vertically and laterally to measure interaction forces within the interfacial volume. A fast-sinusoidal modulation was superimposed on the tip's z-position during image acquisition. As the tip performed linear XY scans, Δf signals were recorded at each position while maintaining the average tip–sample distance by controlling the Δf set-point. By compiling these real-time force measurements across all spatial coordinates, a detailed 3D Δf map with high-spatial resolution representing the local force field near the interface was constructed with nanoscale precision.

The cantilevers were driven photothermally at their resonance frequency with oscillation amplitudes set in the range of 0.1 to 0.2 nm to resolve the distribution of local hydration layers. The frequency and amplitude range of the *z* modulation signal during the 3D-AFM force

mapping were: 195.3 Hz and 5 nm, respectively. On a selected surface area, a 3D-Δf map covering an area of 50—100 x 50—100 nm square—divided into grids of 128 x 128 pixels—was acquired by recording in real time the Δf with respect to the tip positions in 3D interfacial space. The z data of the 3D map includes 256 pixels. Note that for a stable force mapping process, in some cases, we used the dissipation channel as a feedback signal to adjust tip-sample separations.

Computational details

Eighteen AnxA5 molecules (PDB code: 6K22) were arranged to form p6 symmetry structure, [5] which was then solvated by 241,548 water molecules, 780 Na^+, 70 Ca^{2+} (including 54 Ca^{2+} ions in the crystallographic structure), and 656 Cl^-. Amber ff19SB potential[6] was employed for AnxA5, and the SPC/E model[7] was used for water. The parameters developed by Jungwirth *et al.* were employed for Na^+,[8] Ca^{2+}, and Cl−ions [9] to account for the electronic continuum correction, which was also used in the previous MD simulation study of AnxA5.[10] All MD simulations were performed using AMBER22. Periodic boundary conditions were imposed. Long-range interactions were calculated using the particle mesh Ewald method [11] with a 10-Å real-space cut-off. The bonds, including those for the H atoms, were constrained using the SHAKE algorithm[12] to enable a time step of 2 fs.

The system was equilibrated for 30 ns, while AnxA5 and Ca^{2+} ions in the crystallographic structure were restrained to the initial position with a harmonic constraint of 2 kcal/mol/Å^2. The temperature was kept at 300 K using the Berendsen's thermostat.[13] The pressure was maintained at 1 bar using the Monte Carlo barostat, in which the box scale only along z-direction was allowed to change, where a sheet of annexin 5 lays on an xy plain. In the last 10 ns, residues facing membrane (residue number 30–33, 70–79, 98–108, 140–150, 187, 188, 229–233, 260–267, and 303–306) and Ca^{2+} ions in the crystallographic structure were weakly restrained to the initial position with a harmonic constraint of 0.4 kcal/mol/Å^2. This weak

constraint continued to be used in the production run. The resultant dimension of a simulation box was about 306.57 × 177.00 × 149.65 $Å^3$, in which the ionic concentration in the bulk is approximately 1 mM CaCl + 150 mM NaCl. In the production run, simulation was performed using the NVT ensemble at 300 K with the Berendsen's thermostat[13] for 200 ns. The configuration was saved at every 0.1 ps in the first 30 ns, and 300,000 configurations were generated and used to compute three-dimensional water density. To rotate the non-p6 trimer by 60 degrees, the position to constrain membrane facing residues were rotated by its center by 10 degrees and equilibrated for 1 ns. Repeating this procedure 6 times, the non-p6 trimer was rotated (Supplementary Video 7; Figure S10). Three-dimensional water density distribution for the rotated conformation was computed by the same manner as above.

The interaction energies (E) were calculated from the MD trajectories as the total non-bonded interaction energy—comprising electrostatic and van der Waals contributions—via the following equation:

$$E = \sum_i \sum_j 4\varepsilon_{ij} \left\{ \left(\frac{\sigma_{ij}}{r_{ij}} \right)^{12} - \left(\frac{\sigma_{ij}}{r_{ij}} \right)^{6} \right\} + \frac{1}{4\pi\varepsilon_0} \frac{q_i q_j}{r_{ij}}$$

where ε_{ij} and σ_{ij} are the Lennard-Jones parameter between atom i and j, r_{ij} is the distance between atom i and j, ε_0 is the vacuum permittivity, r_i and r_j are charges on atom i and j, respectively. When computing $E_{\text{III-IV}}$, i runs all atoms in domain III and j runs all atoms in domain IV. When computing $E_{\text{III-water}}$, i runs all atoms in domain III and j runs all atoms in N water molecules from the center between domain III and IV. Similarly, when computing $E_{\text{IV-water}}$, i runs all atoms in domain IV and j runs all atoms in N water molecules from the center between domain III and IV. The cases of N = 10, 20, 50, 100, 300, 1000, and 2000 were estimated. The water molecules where their oxygen is within 3.5 Å from any protein atoms were used to calculate the energy in the primary hydration shell. The analysis included direct interactions among interface residues, as well as interactions between these residues and

surrounding water molecules within the hydration shells. The resulting energies were used for comparative analysis of the three junction configurations.

2. Supplementary Figures

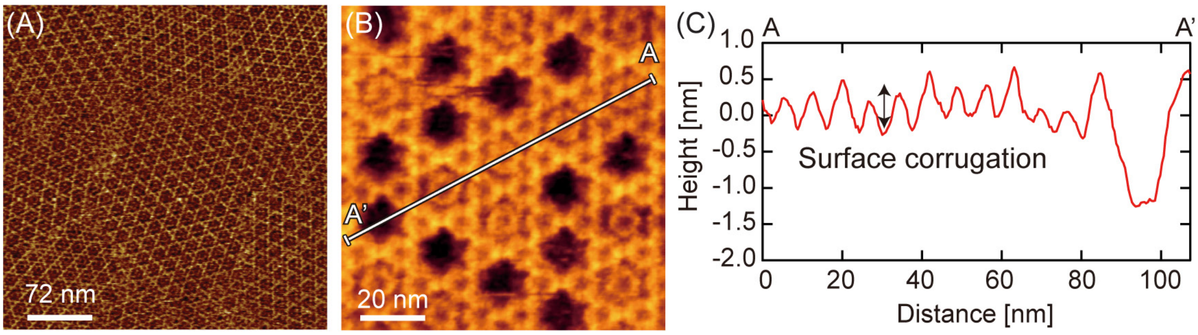

Figure S1. Molecular Arrangement of AnxA5 Crystalline Assembly. (A) High-resolution AFM image of the 2D AnxA5 crystalline array, obtained in the presence of 2 mM Ca^{2+}. AFM image acquired over larger areas indicate that AnxA5 assembles into crystalline lattices comprising domains with distinct orientations, separated by grain boundaries exhibiting relatively lower molecular order. (B) 2D AFM image of AnxA5 assemblies and (C) the height profile, showing the molecular corrugation of about 5 Å. This low roughness indicates that the extended features observed in the 3D-AFM maps correspond to the hydration architecture rather than the surface topographic variations.

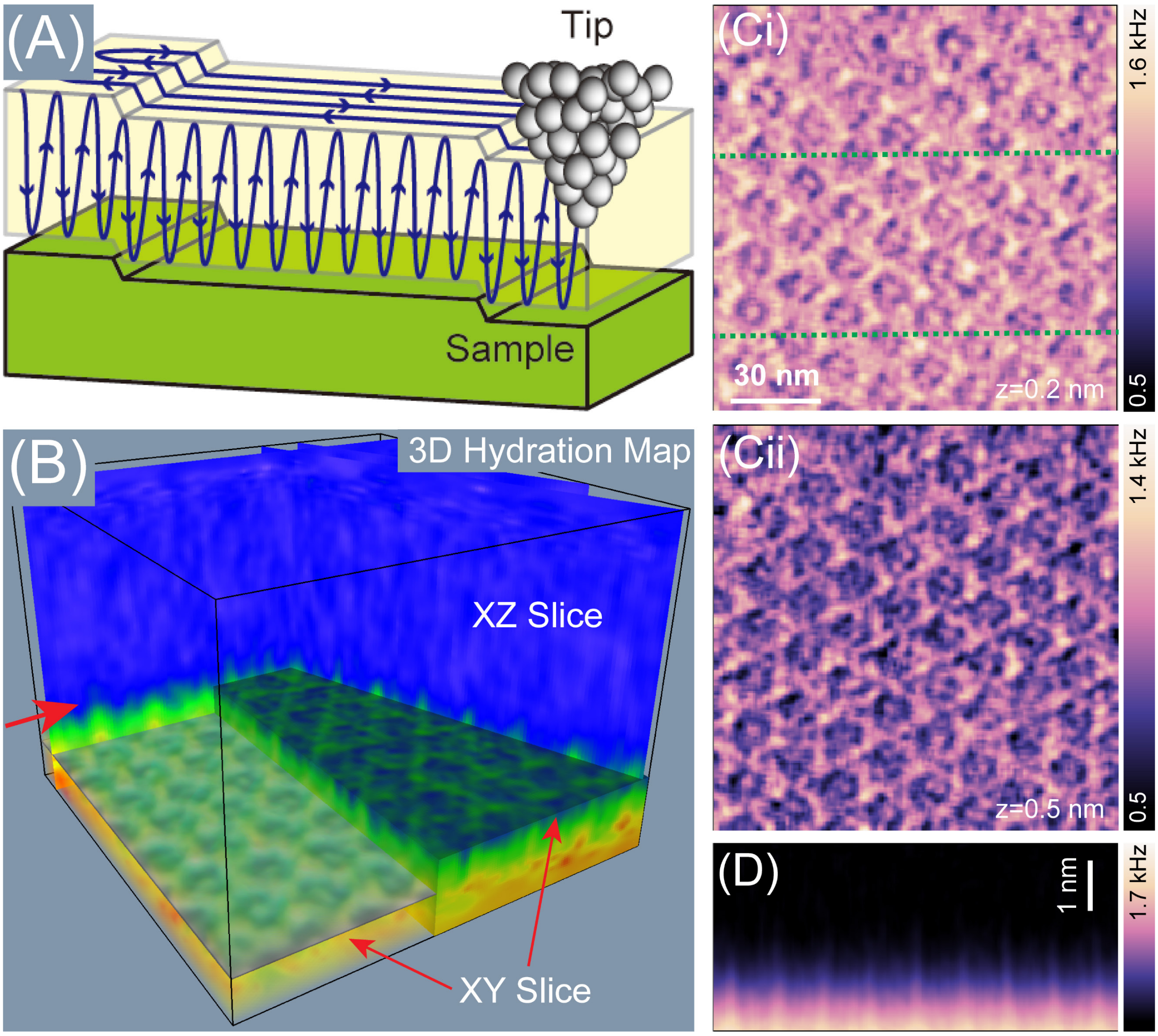

Figure S2: 3D-AFM Maps of Interfacial Structures. (A) Schematic of the 3D-AFM method. The tip executes combined vertical and lateral raster scans to map force interactions throughout the 3D interface, with z-position modulated by a fast sinusoidal signal during lateral motion. **(B)** Representative 3D-AFM volumetric map of interfacial hydration structures surrounding Annexin A5 (AnxA5) protein assemblies on a lipid membrane. XY and XZ slices are shown within the 3D volume. **(Ci–ii)** 2D XY in-plane organization of water at different z-heights, extracted at z = 0.2 nm and z = 0.5 nm, respectively. **(D)** The 2D XZ map, averaged over the region delineated by green dashed lines in panel Ci, reveals the vertical organization of interfacial water. An extended hydration column spanning ~1.5–2 nm is evident.

As illustrated in the 2D vertical (XZ) map (Fig. D), the interface is characterized by a robust hydration column extending approximately 1.5–2 nm into the bulk phase. This spatial organization extends beyond the primary hydration layer, indicating long-range propagation of surface-induced ordering. Such an extension of the hydration zone—comprising roughly six to seven molecular tiers—indicates significant suppression of molecular entropy near the interface. This structured boundary zone, spanning ~2 nm continuously in z while discretely patterned laterally, likely governs interfacial rheology and establishes a steric hydration barrier against both non-specific and specific adsorption. While non-specific interactions are largely suppressed, specific adsorption occurs selectively at interfacial domains characterized by

reduced hydration thickness or localized structural disruptions. The~1.5–2 nm thick hydration gradient implies a significant dehydration penalty for approaching ligands or ions, fundamentally governing the kinetics of adsorption and molecular recognition at the interface.[14]

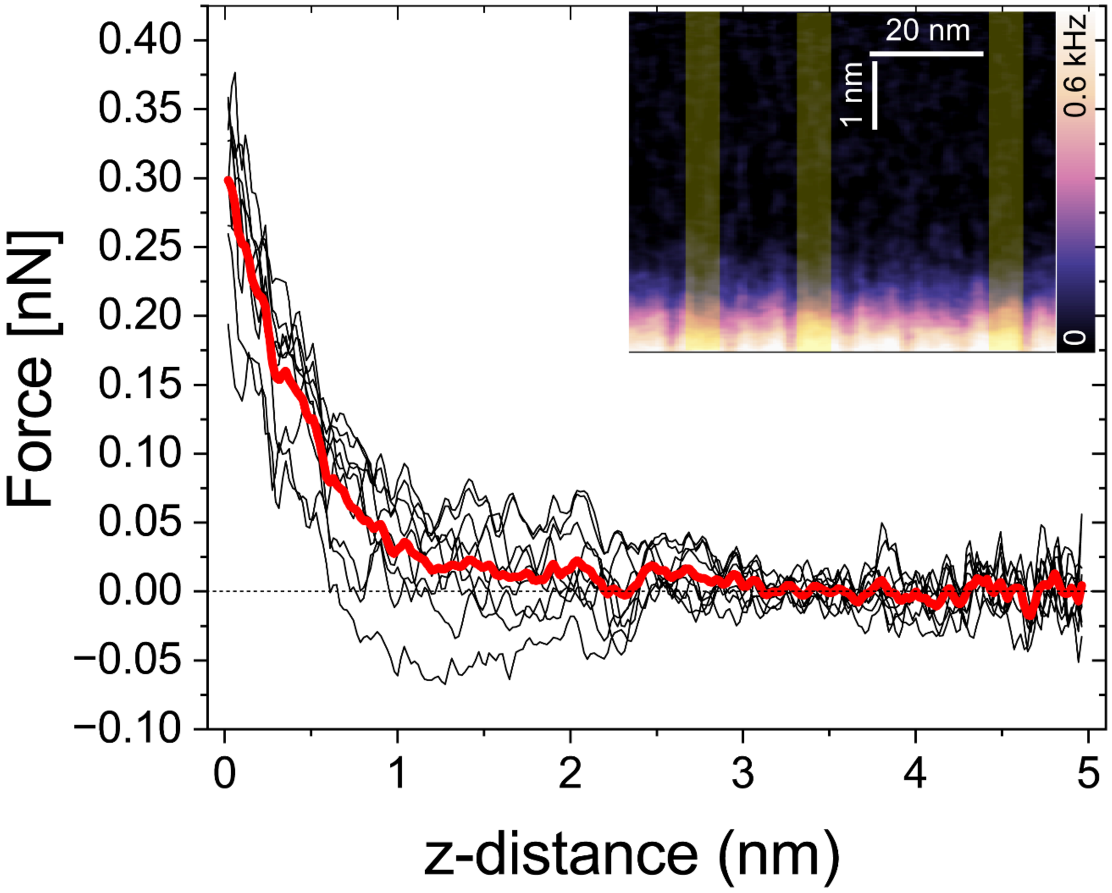

Figure S3. Characteristic of Hydration Forces. Force–distance (F–z) curves were acquired along the shaded region indicated in the inset. The individual force profiles (thin black lines) and their average (thick red line) are shown as a function of distance z from the AnxA5 surface. Unlike the oscillatory force behavior characteristic of ordered hydration structures on solid substrates, the force curves above the protein exhibit a predominantly repulsive profile, corresponding to the hydration layer surrounding the protein. The effective interaction range of this hydration repulsion is approximately 1.5–2.1 nm. This structured hydration zone, characterized by a gradient spanning ~1.5–2.1 nm, implies a significant dehydration penalty for approaching ligands or ions, fundamentally governing the kinetics of adsorption and molecular recognition at the interface.

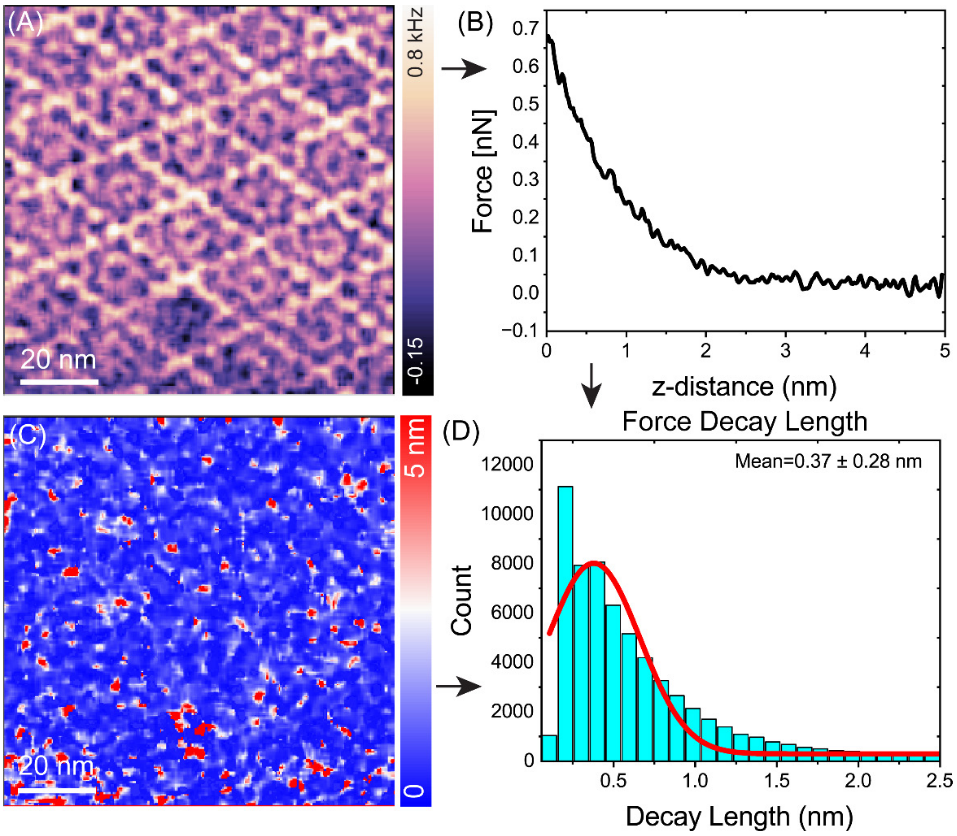

Figure S4. Spatial Distribution of Short-Range Hydration Force Decay Lengths. (A) Two-dimensional XY map of the hydration structure acquired at z=0.5 nm. **(B)** Representative force–distance curve obtained over the hydration structure as a function of vertical displacement. The force was calculated from the frequency shift (Δf) using the Sader method. **(C)** Spatial map of force decay lengths, determined by fitting individual force curves to an exponential decay function, $F(d)=F_0 e^{-d/\lambda}$. **(D)** Histogram showing the distribution of decay lengths extracted from the map shown in (C).

Supplementary Note: To distinguish the short-range signal (which contribute the observed hydration features) from longer-range tip–sample interactions (e.g., electrostatic double-layer or van der Waals interactions), we analyzed the decay length of the 3D-AFM forces that generating the observed hydration pattern. Fitting the non-oscillatory repulsive force branch to an exponential decaying function, $F(d)=F_0 e^{-d/\lambda}$, yields a decay length of ~0.3 nm (0.37 ± 0.28 nm), comparable to a single water layer. The force attenuation occurs over a characteristic length of ~1.0-1.2 nm, matching the hydration region and far shorter than van der Waals or electrostatic decays (~5–10 nm). This short decay confines the signal to the near-surface hydration structure. This identifies the origin of the 3D AFM force pattern as a short-range hydration force (non-DLVO force), rather than a diffuse long-range electrostatic interaction.

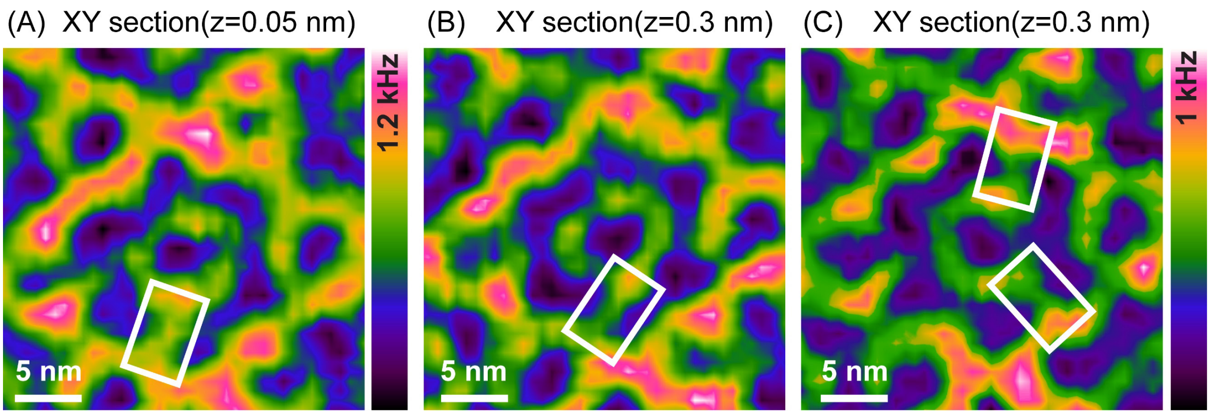

Figure S5. Hydration Structures at the AnxA5 Domain III–IV Junction Linking Central and Crystalline Trimers. (A–C) Two-dimensional planar maps revealing the lateral organization of water molecules at different z-heights above the Annexin A5 (AnxA5) crystalline lattice. Data are extracted at heights of z=0.05 nm (A) and z=0.3 nm **(B–C)** relative to the protein assembly surface. White rectangles indicate the interfacial regions where water-mediated hydrogen-bonding networks are established between the dynamic central trimer and the surrounding crystalline lattice, i.e., at the III-IV junction. These localized hydration structures act as structural bridges, potentially modulating the rotational freedom of the central trimer through specific hydration-shell stabilization.

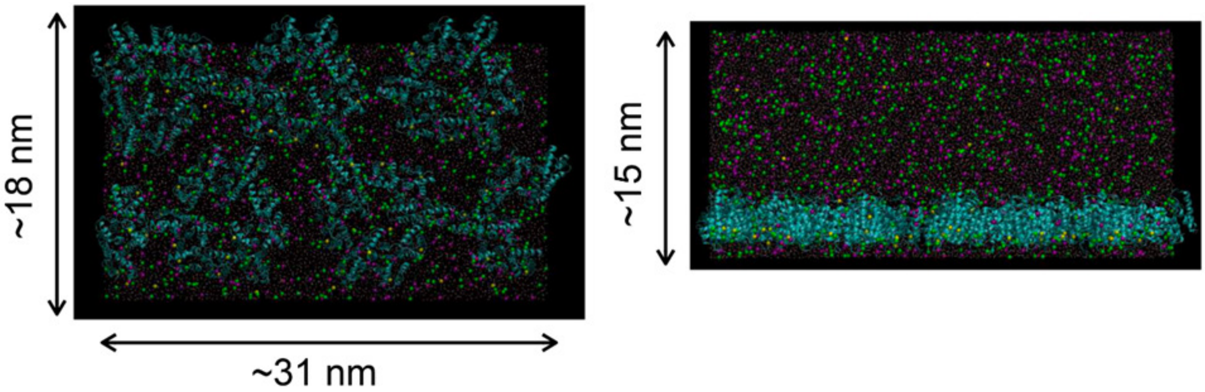

Figure S6. p6 Unit Cell of Simulated AnxA5 Crystalline Lattice. (A) Top view; and **(B)** side view. Ca^{2+} ions are shown as yellow spheres, Na^{+} ions as green and Cl^{-} ions as purple spheres. The simulation was performed in the presence of 1 mM $CaCl_2$, 150 mM NaCl, and water, comprising a total of 816,258 atoms.

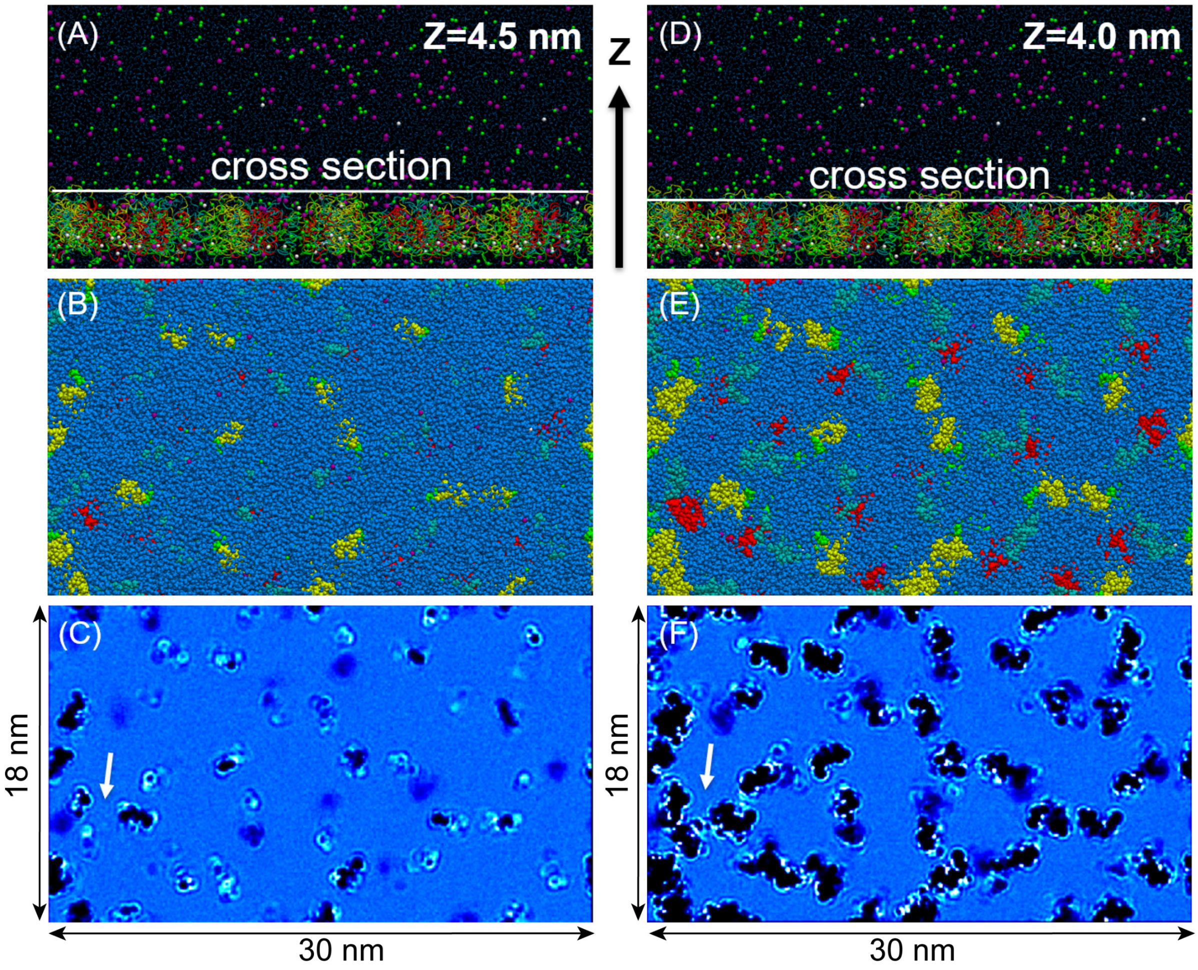

Figure S7. Cross-Sectional and Top Views of AnxA5 Assembly and Interfacial Water Organization. (A) Side-view cross-section of the AnxA5 layer in the presence of Ca^{2+}, Na^{+}, Cl^{-}, and water molecules. **(B)** Top views of the assembled structure. **(C)** Corresponding 2D XY-plane water oxygen density distribution and organization across the surface at height z = 4.5 nm. **(D–F)** As in (A–C), with density extracted at height z = 4.0 nm. The white arrow highlights the domain III–IV boundary between crystalline and non-crystalline trimers.

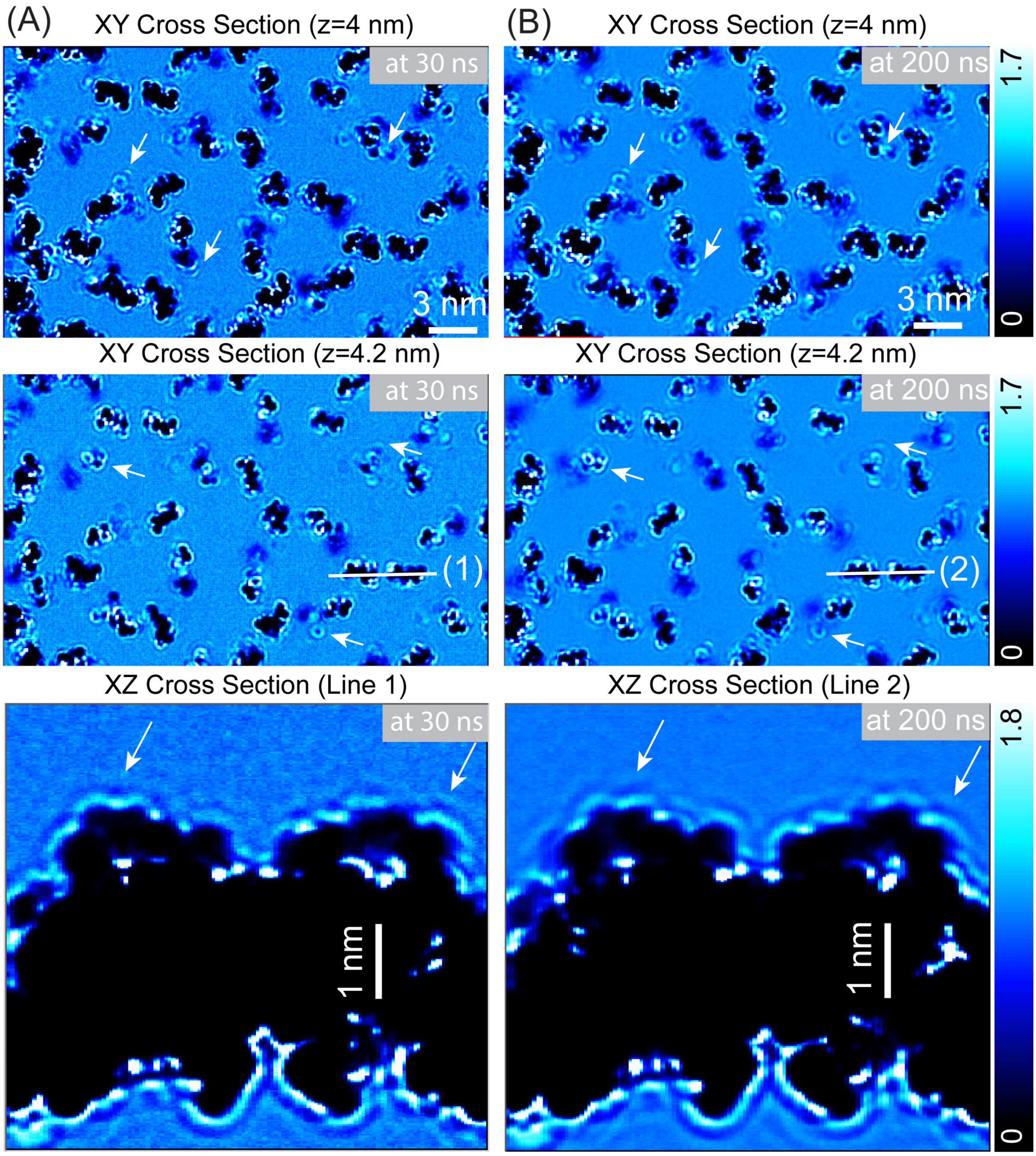

Figure S8. Interfacial Water Structure: Comparison of 30 ns and 200 ns Sampling Windows. Two-dimensional XY-plane water oxygen density maps and spatial organization across the surface at heights z=4.0 and 4.2 nm, determined at 30 ns **(A)** and 200 ns **(B).** Corresponding vertical XZ profiles are shown along Line (1) for 30 ns and Line (2) for 200 ns. White arrows highlight identical interfacial features. The similarity in water oxygen density patterns clearly indicates that the system has already reached equilibrium by 30 ns.

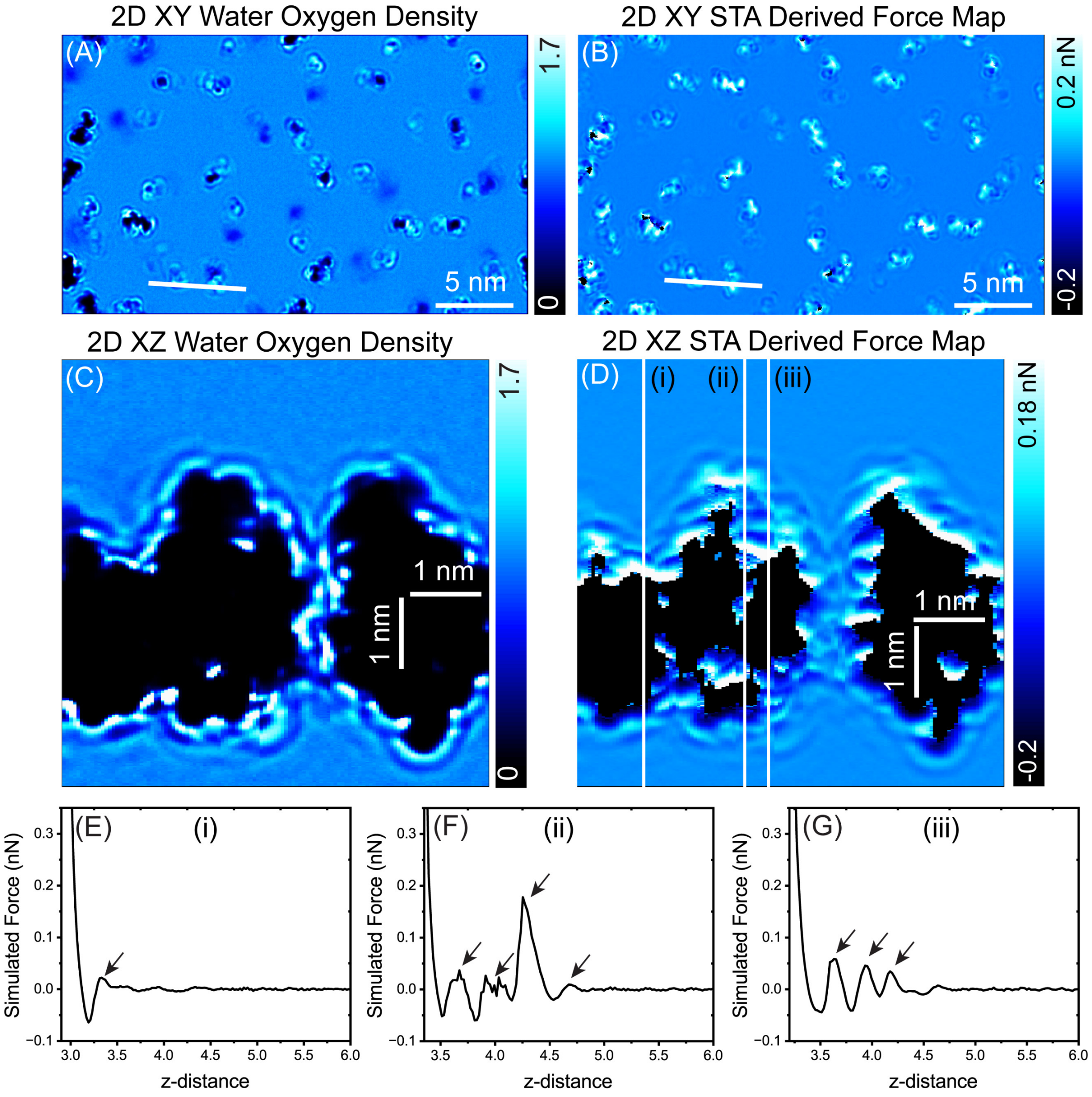

Figure S9. Simulated Force Maps and Representative Force–Distance Curves Derived from MD Water Density Distributions Using Solvent Tip Approximation (STA). (A) Two-dimensional (2D) *XY* spatial distribution of water oxygen density above the protein assembly. **(B)** STA-derived force map, obtained using the formula: $F_z(z)=k_B T(1/\rho(z))\partial\rho(z)/\partial z$. **(C)** 2D vertical cross-section of the water oxygen density along white line in (A), and **(D)** the corresponding force map. **(E–G)** Three representative individual force-distance curves extracted along the vertical trajectories denoted as (i), (ii), and (iii) in panel (D). Single or multiple distinct force peaks, indicative of discrete water layering, are clearly resolved. In certain instances, such as in panel (E), a weak force peak alongside an attractive branch is also observed. This comparison highlights the pronounced nanoscale spatial heterogeneity of the hydration shell at the protein surface. Although MD simulations resolve these highly localized solvation structures, the temporal and spatial averaging inherent to AFM measurements likely convolves these distinct features into the broader repulsive profile observed experimentally **(Figure S3).**

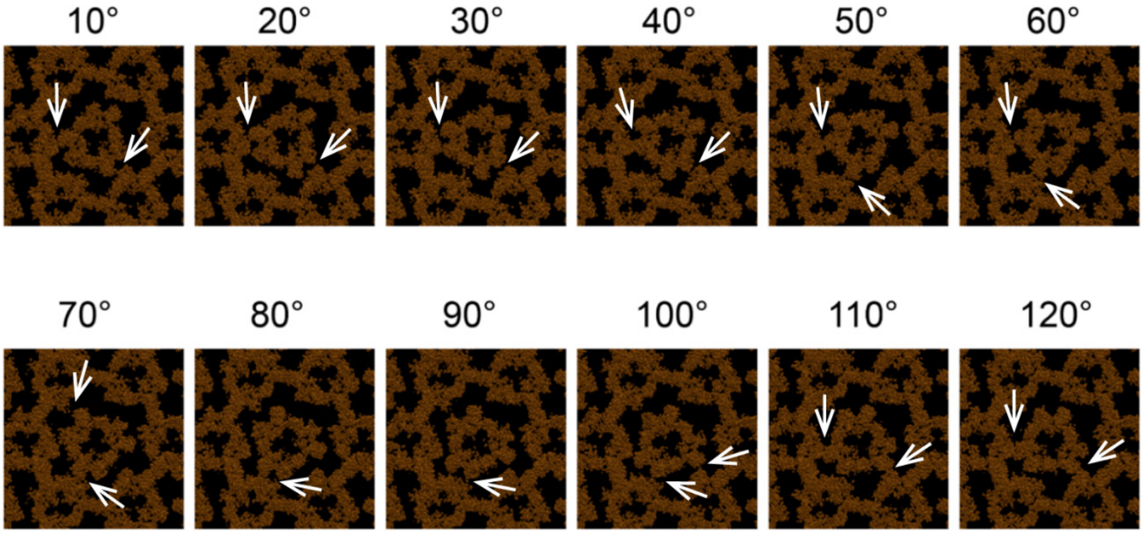

Figure S10: Central Trimer Rotation within the *p6*-Crystalline Assembly. A series of structural snapshots of the Annexin A5 2D lattice illustrating the stepwise rotation of the central trimer at discrete angles ranging from 10° to 120°, while the surrounding trimers in the *p6*-crystalline lattice remain fixed in their original configuration. The white arrows indicate the close-contact arrangements between the crystalline and non-crystalline trimers.

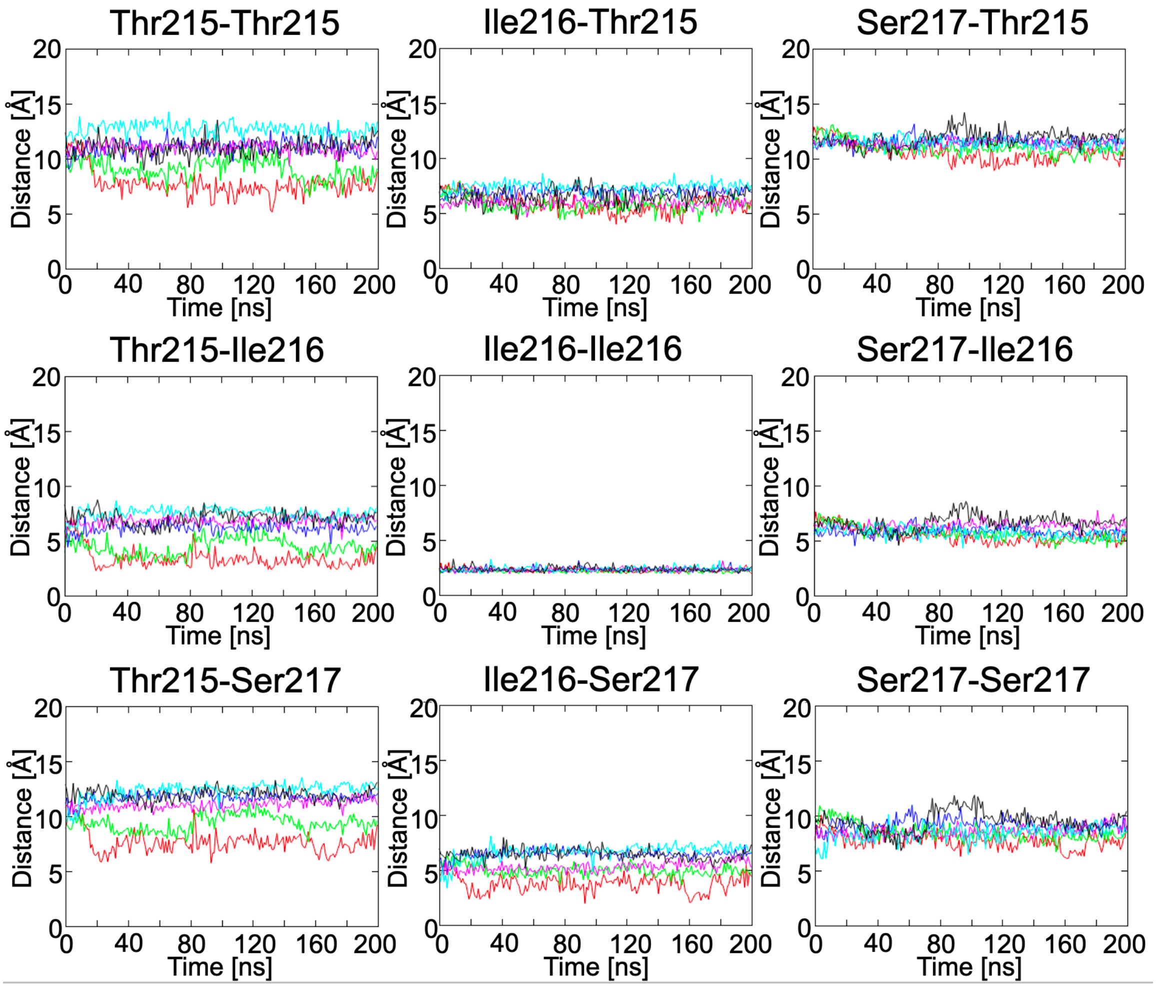

Figure S11. Time Evolution of Proximal Residue Distances at Neighboring Domain III–III Interfaces among Six Trimers in the Crystalline Assembly. The junction region involves the following residue pairs for each set of adjacent trimers: Thr215–Thr215, Thr215–Ile216, Thr215–Ser217, Ile216–Thr215, Ile216–Ile216, Ile216–Ser217, Ser217–Thr215, Ser217–Ile216, and Ser217–Ser217. Among these, the Ile216–Ile216 contacts exhibit the shortest separations, with minimum distances of approximately 3 Å, indicative of tight packing at this interface. The remaining inter-residue distances range from ~6 to 12 Å, with more extended contacts predominantly observed for Thr215–Thr215, Thr215–Ser217, Ser217–Thr215, and Ser217–Ser217 pairs, which display separations of ~9–12 Å. Each color represents one of the six distinct domain III–III junctions between neighboring trimeric units in the hexameric lattice.

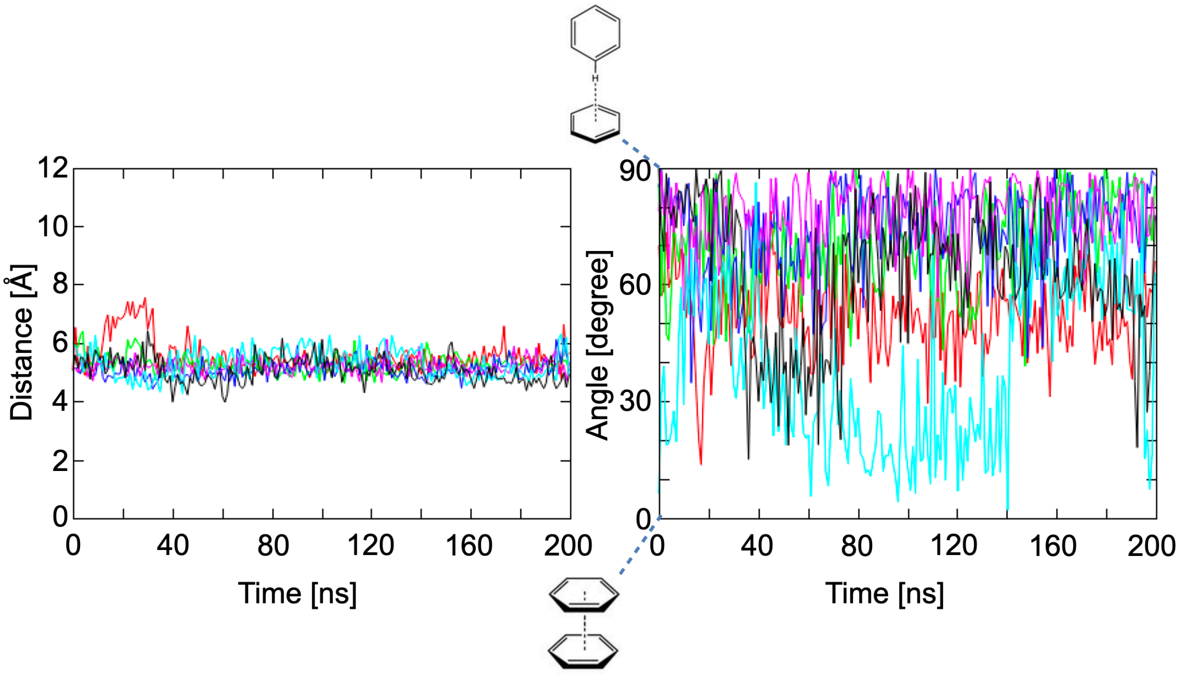

Figure S12. Minimum Phe180–Phe180 Distances at Domain III–III Interfaces across the Six Trimer Junctions in the Crystalline Assembly. The closest contacts, measured at approximately 5.5–6 Å across all junctions, are consistent with aromatic π–π interactions. These π–π contacts include both edge-to-face (T-shaped) and nearly parallel (face-to-face) geometries, highlighting the diverse stacking modes and conformational flexibility of aromatic residues that contribute to the stabilization of the Annexin A5 2D lattice.

Supplementary Note: The residues Thr215, Ile216, Ser217, and Phe180 in domain III of AnxA5 are positioned at the inter-domain interface and likely contribute to stabilizing interactions between adjacent crystalline trimers. Within the assembled 2D lattice, the inter-residue distances among these pairs range from 3 to 12 Å, predominantly between 6 and 12 Å. Among them, Ile216–Ile216 pairs form the closest contacts at six trimer–trimer junctions, with minimum separations of approximately 3 Å (**Fig. S11; Supplementary Video 8**). The remaining inter-residue separations, including Thr215–Thr215, Thr215–Ser217, Ser217–Thr215, and Ser217–Ser217 pairs, span roughly 9–12 Å (**Fig. S11**). In contrast, Phe180–Phe180 interactions occur at distances of about 5 Å, consistent with aromatic π–π stacking that involves both T-shaped (edge-to-face) and nearly parallel (face-to-face) configurations (**Fig. S12; Supplementary Video 9**).

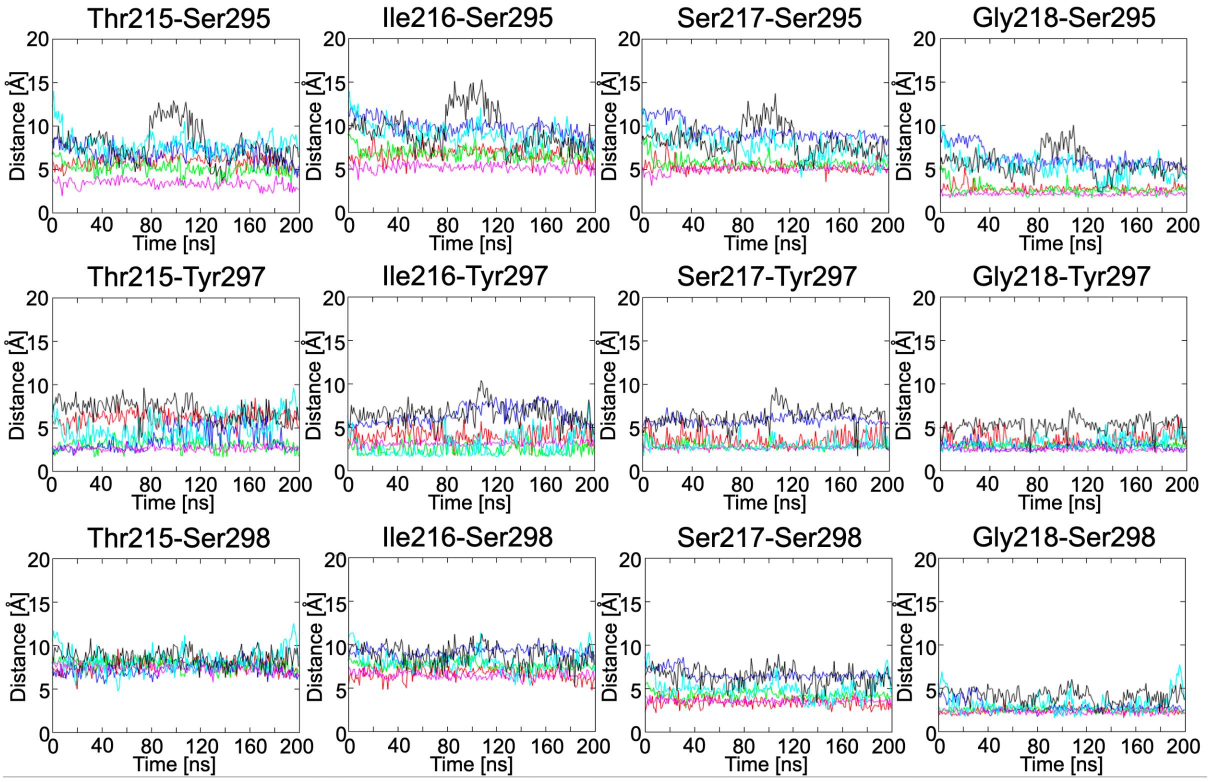

Figure S13. Minimum Inter-Residue Distances at Neighboring Domain III–IV Interfaces between Crystalline and Non-Crystalline Regions. For each of the six distinct junctions formed between the rotating non-crystalline trimer and the adjacent crystalline trimer, the minimum distance for every amino acid pair is shown, including Thr215–Ser295, Thr215–Tyr297, Thr215–Ser298, Ile216–Ser295, Ile216–Tyr297, Ile216–Ser298, Ser217–Tyr297, Gly218–Ser295, Gly218–Tyr297, and Gly218–Ser298. Across all junctions, the shortest inter-residue separations are generally below 12 Å, with several pairs—most notably Gly218–Ser298, Gly218–Tyr297, Ser217–Tyr297, and Ile216–Tyr297—exhibiting distances of 5 Å or less. Overall, many residues at the III–IV interfaces approach each other more closely than those at the purely crystalline III–III interfaces, although residues involved in the III–III contacts exhibit more favorable mutual orientations, likely contributing to the enhanced energetic stability of the crystalline lattice. Each color denotes one of the six distinct domain III–IV junctions between the non-crystalline trimer and its neighboring trimeric units in the hexameric assembly.

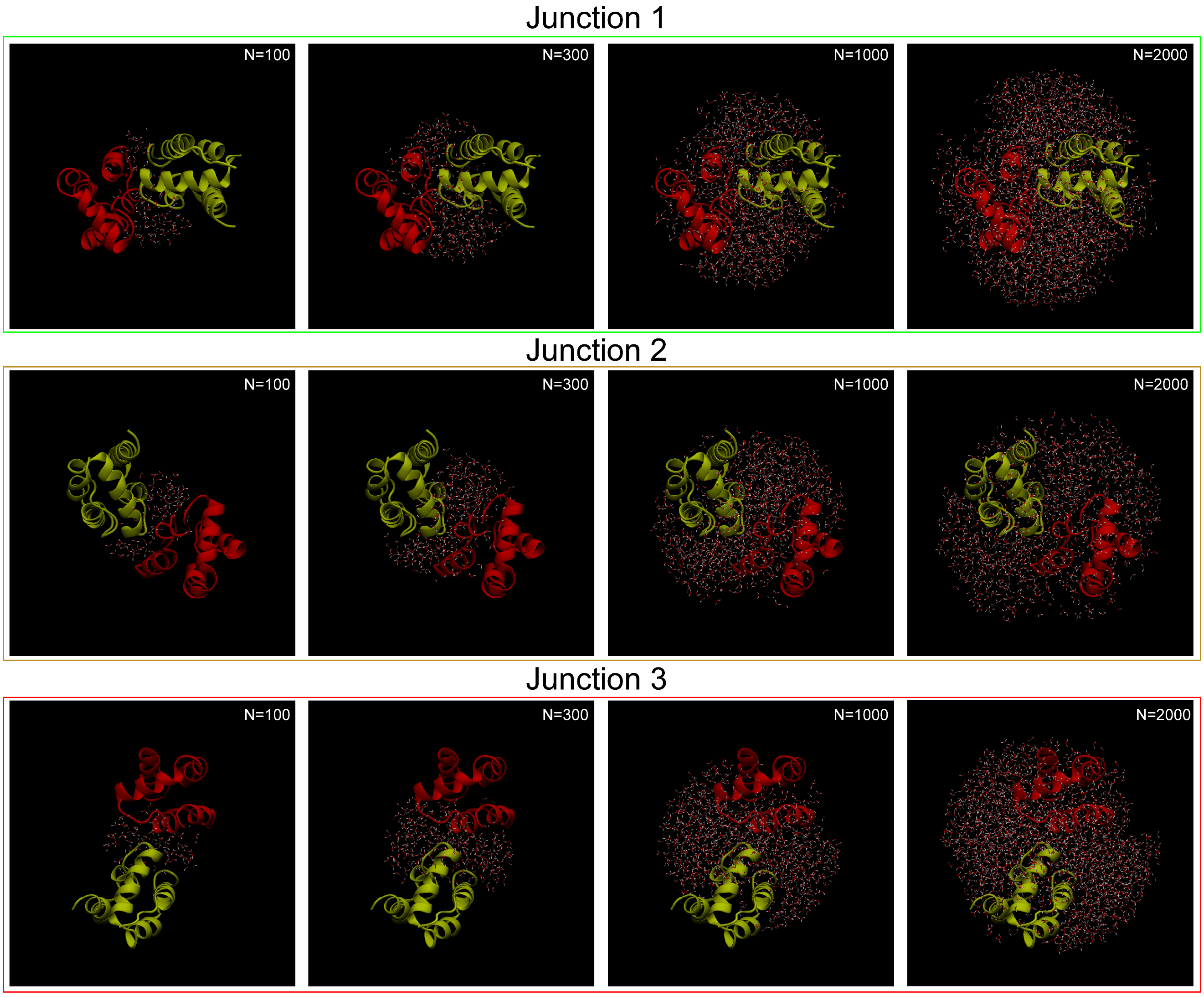

Figure S14: Schematic of Junction III–IV, showing the three contacts and intervening water organization used to calculate non-bonded interaction energies for systems involving N=100, 300, 1000, and 2000 water molecules.

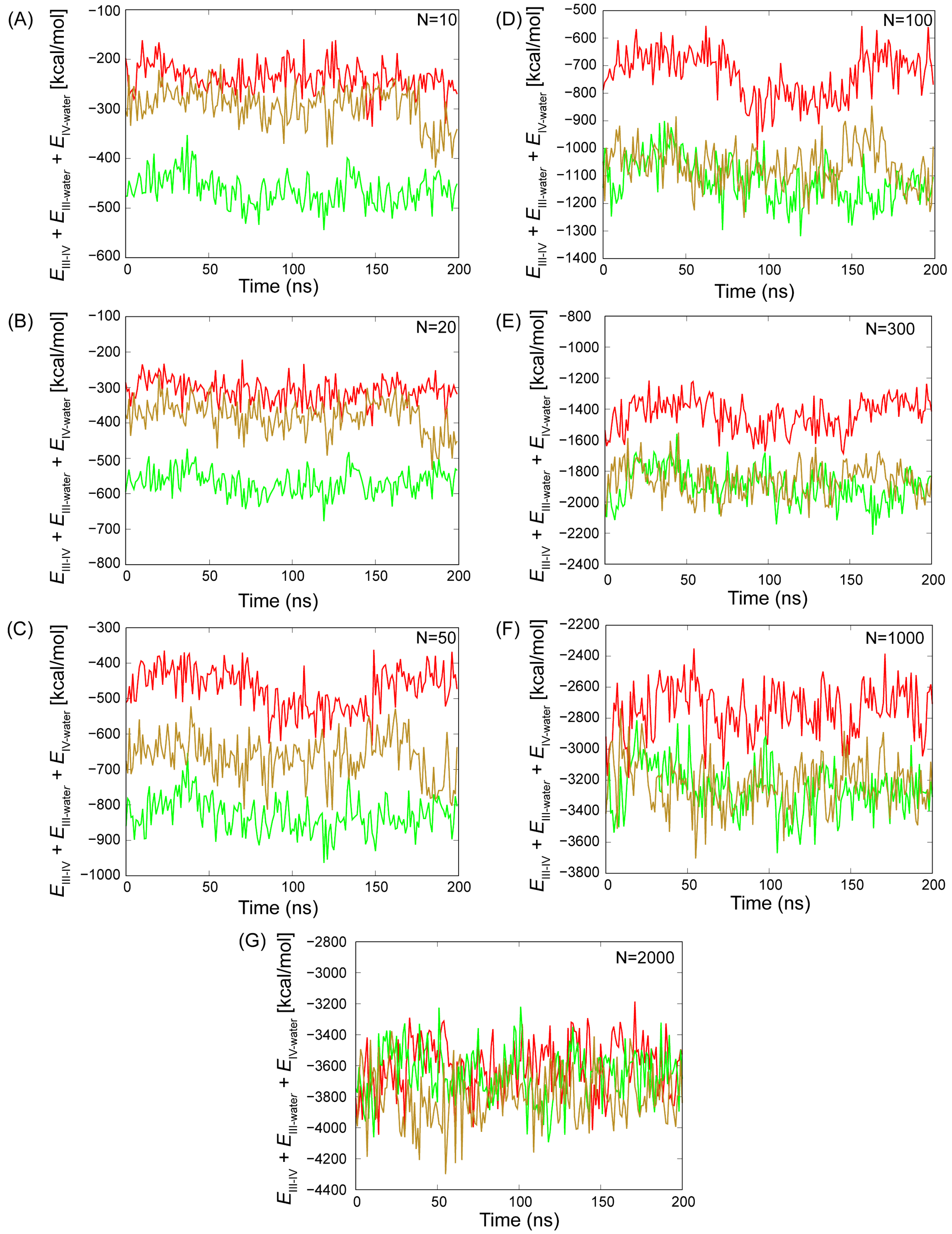

Figure S15. Convergence of Interaction Energy Profiles with Increasing Interfacial Water Content. Comparative plots of interaction energies for Junctions 1, 2, and 3 calculated with increasing numbers of interfacial water molecules: **(A)** N=10, **(B)** N=20, **(C)** N=50, **(D)** N=100, **(E)** N=300, **(F)** N=1000, and **(G)** N=2000. At low water content (N<100), the energy profiles for Junctions 1 and 2 are distinct. As the solvation shell expands, a distinct convergence in energy levels between Junction 1 and Junction 2 is observed. At N = 2000 molecules, the

energetic profiles of Junctions 1, 2, and 3 converge. Although the absolute energy magnitudes scale with N, the characteristic energetic degeneracy (the minimal energy difference) between Junction 1 and Junction 2 stabilizes and remains consistent from N=100 to N=1000. This confirms that the essential features of the hydration-mediated energy landscape are effectively captured within a local solvation shell of approximately 100–300 water molecules.

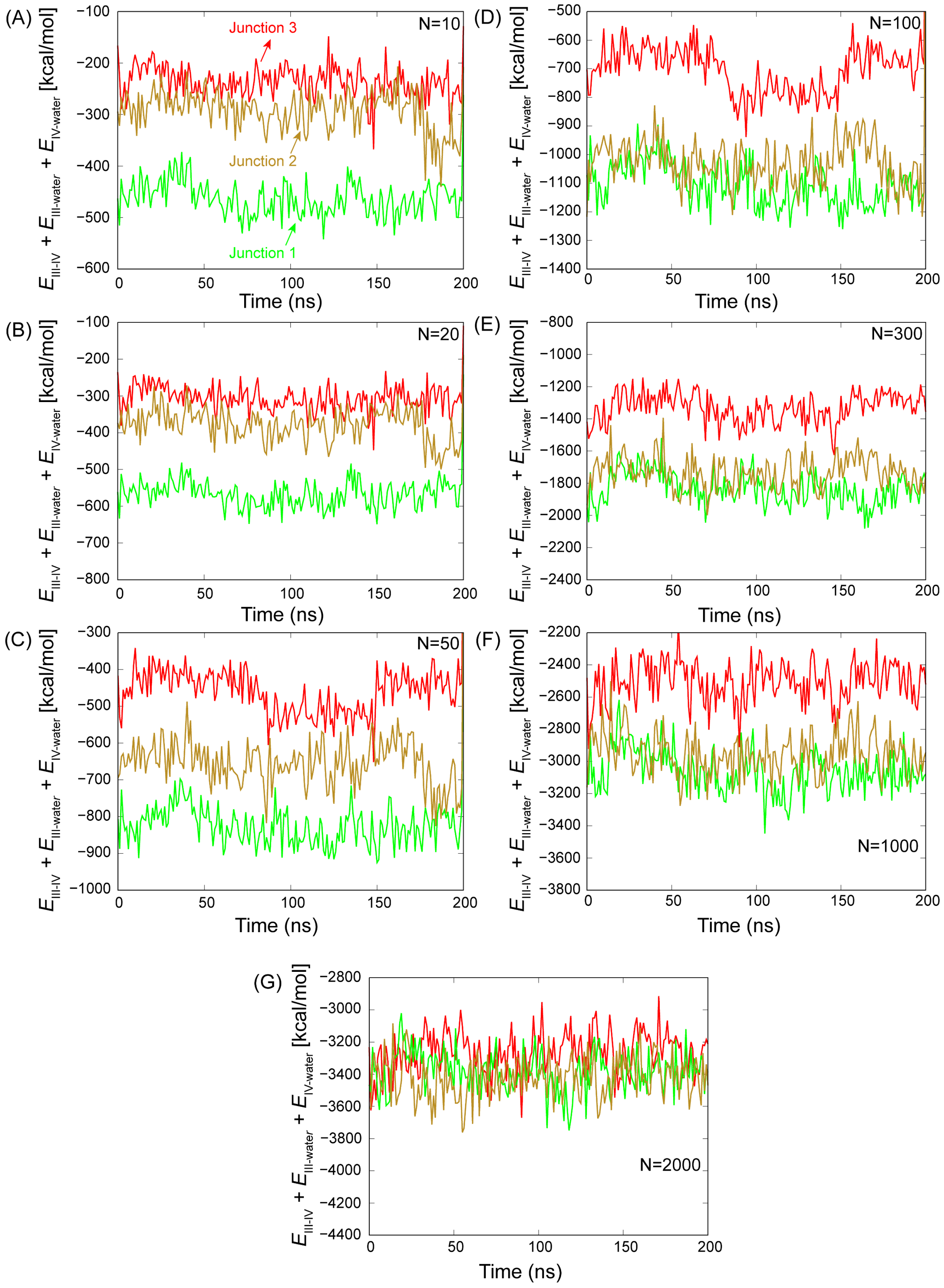

Figure S16. Interfacial Interaction Energy Profiles for the Three Junction Configurations Formed between Domains III and IV, Evaluated Considering Only Water Molecules within the First Hydration Shell. Energy profiles for Junction 1 (green), Junction 2 (dark yellow), and Junction 3 (red) were calculated under varying total water populations: **(A)** N=10, **(B)** N=20, **(C)** N=50, **(D)** N=100, **(E)** N=300, **(F)** N=1000, and **(G)** N=2000 water molecules. The calculated energies include contributions from both direct residue–residue interactions

between Domains III and IV and their interactions with water molecules confined to the first hydration shell. At low hydration levels ($N \leq 50$), nearly all water molecules are recruited into the primary hydration shell. As the solvent domain expands, the first hydration shell accommodates approximately 80, 180, 400, and 490 molecules for systems containing $N = 100$, 300, 1000, and 2000 total water molecules, respectively. At $N = 2000$ molecules, the energetic profiles of Junctions 1, 2, and 3 converge, indicating stabilization of junction energetics upon establishment of the local solvent environment. Notably, excluding higher-order hydration shells yields no significant deviation in interaction energies compared to fully solvated models (Figure S15). This demonstrates the short-range dominance of interfacial interactions, confirming that the energy landscape is governed almost exclusively by direct inter-domain residue contacts and the immediate localized solvent shells.

3. Supplementary Movies

Supplementary Video 1. Side view of AnxA5 in the assembled two-dimensional lattice, highlighting the dynamically fluctuating N-terminal domains protruding from the top surface of the protein layer, opposite to the Ca^{2+}-binding interface. [MP4, 5.35 MB]

Supplementary Video 2. Side view of AnxA5 assembled into a two-dimensional lattice, highlighting the C-terminal domains. [MP4, 5.01 MB]

Supplementary Video 3. Interfacial water density distribution near the AnxA5 protein assembly at a 30 ns simulation window. Two-dimensional XY-plane maps of water oxygen density across the AnxA5 crystalline surface, extracted from the three-dimensional density distribution. **Color:** brighter blue indicates higher water density, darker blue indicates lower density, and black contours represent the protein structure. **Size:** 18 nm x 30 nm. [MP4, 6.32 MB]

Supplementary Video 4. Interfacial water density distribution near the AnxA5 protein assembly at a 200 ns simulation window. Two-dimensional XY-plane maps of water oxygen density across the AnxA5 crystalline surface, extracted from the three-dimensional density distribution. **Color bar:** brighter blue indicates higher water density, darker blue indicates lower density, and black contours represent the protein structure. **Size:** 18 nm x 30 nm. [MP4, 8.88 MB]

Supplementary Video 5. Cross-sectional and top views of the assembled AnxA5 crystal with interfacial water organization. Left: side-view cross-section of the AnxA5 layer in the presence of Ca^{2+}, Na^{+}, Cl^{-}, and water. Right: two-dimensional top view of the assembled surface. Bottom right: water oxygen density distribution across the surface. The video illustrates the interfacial organization at heights **z = 5.5, 5.0, 4.5, 4.0, 3.5, 3.0, and 2.5 nm**. [MP4, 9.18 MB]

Supplementary Video 6. Water oxygen density distribution across the AnxA5 assembly with distinct color coding. Two-dimensional XY-plane maps of water oxygen density across the Annexin A5 crystalline surface, extracted from the three-dimensional density distribution. **Size:** 18 × 30 nm. [MOV, 4.65 MB]

Supplementary Video 7. Rotational motion of the central non-crystalline p6 trimer in the 2D AnxA5 assembly. [MP4, 7.38 MB]

Supplementary Video 8. Visualization of the interfacial contact region between neighboring domain III–III interfaces, highlighting the water molecules located between interacting Ile–Ile residue pairs. [MP4, 1.33 MB]

Supplementary Video 9. Visualization of the interfacial contact region between neighboring domain III–III interfaces, highlighting the edge-to-face (T-shaped) π–π stacking interaction between aromatic Phe–Phe residue pairs. [MP4, 1.19 MB]

4. References

(1) Yamada, R.; Trang, T. N.; Flechsig, H.; Takeda, T.; Kodera, N.; Konno, H. Importance of annexin V N-terminus for 2D crystal formation and quick purification protocol of recombinant annexin V. *PLoS One* **2022**, *17* (12), e0278553.
(2) Miyagi, A.; Chipot, C.; Rangl, M.; Scheuring, S. High-speed atomic force microscopy shows that annexin V stabilizes membranes on the second timescale. *Nature Nanotechnology* **2016**, *11* (9), 783-790.
(3) Fukuma, T.; Kimura, M.; Kobayashi, K.; Matsushige, K.; Yamada, H. Development of low noise cantilever deflection sensor for multienvironment frequency-modulation atomic force microscopy. *Rev. Sci. Instrum.* **2005**, *76* (5), 053704.
(4) Fukuma, T.; Garcia, R. Atomic- and Molecular-Resolution Mapping of Solid–Liquid Interfaces by 3D Atomic Force Microscopy. *ACS Nano* **2018**, *12* (12), 11785.
(5) Hong, S.; Na, S.; Kim, O.-H.; Jeong, S.; Oh, B.-C.; Ha, N.-C. High-resolution structures of annexin A5 in a two-dimensional array. *Journal of Structural Biology* **2020**, *209* (1), 107401.
(6) Tian, C.; Kasavajhala, K.; Belfon, K. A. A.; Raguette, L.; Huang, H.; Migues, A. N.; Bickel, J.; Wang, Y.; Pincay, J.; Wu, Q.; et al. ff19SB: Amino-Acid-Specific Protein Backbone Parameters Trained against Quantum Mechanics Energy Surfaces in Solution. *Journal of Chemical Theory and Computation* **2020**, *16* (1), 528-552.
(7) Berendsen, H. J. C.; Grigera, J. R.; Straatsma, T. P. The missing term in effective pair potentials. *The Journal of Physical Chemistry* **1987**, *91* (24), 6269-6271.
(8) Kohagen, M.; Mason, P. E.; Jungwirth, P. Accounting for Electronic Polarization Effects in Aqueous Sodium Chloride via Molecular Dynamics Aided by Neutron Scattering. *The Journal of Physical Chemistry B* **2016**, *120* (8), 1454-1460.
(9) Martinek, T.; Duboué-Dijon, E.; Timr, Š.; Mason, P. E.; Baxová, K.; Fischer, H. E.; Schmidt, B.; Pluhařová, E.; Jungwirth, P. Calcium ions in aqueous solutions: Accurate force field description aided by ab initio molecular dynamics and neutron scattering. *The Journal of Chemical Physics* **2018**, *148* (22), 222813.
(10) Lin, Y.-C.; Chipot, C.; Scheuring, S. Annexin-V stabilizes membrane defects by inducing lipid phase transition. *Nat. Commun.* **2020**, *11* (1), 230.
(11) Essmann, U.; Perera, L.; Berkowitz, M. L.; Darden, T.; Lee, H.; Pedersen, L. G. A smooth particle mesh Ewald method. *The Journal of Chemical Physics* **1995**, *103* (19), 8577-8593.
(12) Ryckaert, J.-P.; Ciccotti, G.; Berendsen, H. J. C. Numerical integration of the cartesian equations of motion of a system with constraints: molecular dynamics of n-alkanes. *Journal of Computational Physics* **1977**, *23* (3), 327-341.
(13) Berendsen, H. J. C.; Postma, J. P. M.; van Gunsteren, W. F.; DiNola, A.; Haak, J. R. Molecular dynamics with coupling to an external bath. *The Journal of Chemical Physics* **1984**, *81* (8), 3684-3690.
(14) Israelachvili, J. N.; Pashley, R. M. Molecular layering of water at surfaces and origin of repulsive hydration forces. *Nature* **1983**, *306* (5940), 249-250.
(15) Raynal, P.; Pollard, H. B. Annexins: the problem of assessing the biological role for a gene family of multifunctional calcium- and phospholipid-binding proteins. *Biochimica et Biophysica Acta (BBA) - Reviews on Biomembranes* **1994**, *1197* (1), 63-93. Kominami, H.; Hirata, Y.; Yamada, H.; Kobayashi, K. Protein nanoarrays using the annexin A5 two-dimensional crystal on supported lipid bilayers. *Nanoscale Advances* **2023**, *5* (15), 3862-3870.
