# Supplementary material for: Hydration-Mediated Energy Landscapes Govern Rotational Flexibility in Membrane-Bound Annexin V Assemblies
Source: Nano Lett. 2026 Apr 6;26(14):4719–29. doi: 10.1021/acs.nanolett.6c00388 (PMC13088367; doi:10.1021/acs.nanolett.6c00388)
Supplement: Supplementary file 11 [file nl6c00388_si_011.pdf]

Supplementary Video 1. Side view of AnxA5 in the assembled two-dimensional lattice, highlighting the dynamically fluctuating N-terminal domains protruding from the top surface of the protein layer, opposite to the Ca^{2+}-binding interface. [MP4, 5.35 MB]

Supplementary Video 2. Side view of AnxA5 assembled into a two-dimensional lattice, highlighting the C-terminal domains. [MP4, 5.01 MB]

Supplementary Video 3. Interfacial water density distribution near the AnxA5 protein assembly at a 30 ns simulation window. Two-dimensional XY-plane maps of water oxygen density across the AnxA5 crystalline surface, extracted from the three-dimensional density distribution. **Color:** brighter blue indicates higher water density, darker blue indicates lower density, and black contours represent the protein structure. Size: 18 nm x 30 nm. [MP4, 6.32 MB]

Supplementary Video 4. Interfacial water density distribution near the AnxA5 protein assembly at a 200 ns simulation window. Two-dimensional XY-plane maps of water oxygen density across the AnxA5 crystalline surface, extracted from the three-dimensional density distribution. **Color bar:** brighter blue indicates higher water density, darker blue indicates lower density, and black contours represent the protein structure. Size: 18 nm x 30 nm. [MP4, 8.88 MB]

Supplementary Video 5. Cross-sectional and top views of the assembled AnxA5 crystal with interfacial water organization. Left: side-view cross-section of the AnxA5 layer in the presence of Ca^{2+}, Na^{+}, Cl^{-}, and water. Right: two-dimensional top view of the assembled surface. Bottom right: water oxygen density distribution across the surface. The video illustrates the interfacial organization at heights **z = 5.5, 5.0, 4.5, 4.0, 3.5, 3.0, and 2.5 nm**. [MP4, 9.18 MB]

Supplementary Video 6. Water oxygen density distribution across the AnxA5 assembly with distinct color coding. Two-dimensional XY-plane maps of water oxygen density across the **Annexin A5** crystalline surface, extracted from the three-dimensional density distribution. **Size:** 18 × 30 nm. [MOV, 4.65 MB]

Supplementary Video 7. Rotational motion of the central non-crystalline p6 trimer in the 2D AnxA5 assembly. [MP4, 7.38 MB]

Supplementary Video 8. Visualization of the interfacial contact region between neighboring domain III–III interfaces, highlighting the water molecules located between interacting Ile–Ile residue pairs. [MP4, 1.33 MB]

Supplementary Video 9. Visualization of the interfacial contact region between neighboring domain III–III interfaces, highlighting the edge-to-face (T-shaped) π–π stacking interaction between aromatic Phe–Phe residue pairs. [MP4, 1.19 MB]
